# A first step towards a framework for interventions for individual working practice to prevent work-related musculoskeletal disorders: a scoping review

**DOI:** 10.1186/s12891-023-06155-w

**Published:** 2023-02-01

**Authors:** Bert van de Wijdeven, Bart Visser, Joost Daams, Paul P.F.M. Kuijer

**Affiliations:** 1 Public and Occupational Health, Amsterdam UMC location University of Amsterdam, Meibergdreef 9, K0-116 1105 AZ Amsterdam, The Netherlands; 2 Centre of Expertise Urban Vitality, Amsterdam University of Applied Sciences, Amsterdam, The Netherlands

**Keywords:** Work related risk factors, Occupational training, Ergonomic interventions, Musculoskeletal diseases, Prevention and control

## Abstract

**Background:**

Work-related musculoskeletal disorders (WMSDs) are a key topic in occupational health. In the primary prevention of these disorders, interventions to minimize exposure to work-related physical risk factors are widely advocated. Besides interventions aimed at the work organisation and the workplace, interventions are also aimed at the behaviour of workers, the so-called individual working practice (IWP). At the moment, no conceptual framework for interventions for IWP exists. This study is a first step towards such a framework.

**Methods:**

A scoping review was carried out starting with a systematic search in Ovid Medline, Ovid Embase, Ovid APA PsycInfo, and Web of Science. Intervention studies aimed at reducing exposure to physical ergonomic risk factors involving the worker were included. The content of these interventions for IWP was extracted and coded in order to arrive at distinguishing and overarching categories of these interventions for IWP.

**Results:**

More than 12.000 papers were found and 110 intervention studies were included, describing 810 topics for IWP. Eventually eight overarching categories of interventions for IWP were distinguished: (1) Workplace adjustment, (2) Variation, (3) Exercising, (4) Use of aids, (5) Professional skills, (6) Professional manners, (7) Task content & task organisation and (8) Motoric skills.

**Conclusion:**

Eight categories of interventions for IWP are described in the literature. These categories are a starting point for developing and evaluating effective interventions performed by workers to prevent WMSDs. In order to reach consensus on these categories, an international expert consultation is a necessary next step.

**Supplementary Information:**

The online version contains supplementary material available at 10.1186/s12891-023-06155-w.

## Background

Musculoskeletal disorders (MSDs) are defined by the World Health Organisation (WHO) as “health problems of the locomotor apparatus, i.e. muscles, tendons, the skeleton, cartilage, ligaments and nerves. MSDs include all forms of ill-health ranging, from light, transitory disorders to irreversible disabling injuries [1]. An overview in 2013 across 188 countries of the 25 most common causes of “years lived with disability” showed that MSDs are highly prevalent. Top of the list is low back pain, fourth is neck pain, and tenth in that list are “other MSD complaints” [2]. In addition to personal suffering, MSDs also cause direct and indirect economic cost, such as healthcare cost and lost productivity [3]. In Europe the total cost of work-related MSDs due to lost productivity among people of working age is estimated as 2% of the gross domestic product (GDP). In Europe MSDs are responsible for 50% of all absences from work lasting for more than three days and about 60% of all reported cases of permanent incapacity [4]. Worldwide low back pain arising from ergonomic exposures at work was estimated to cause 21.7 million disability-adjusted life years in 2010. These are the years of life lost as a result of premature death plus the years lived with a disability [5].

MSDs induced or aggravated by work and the circumstances of its performance are called work-related MSDs (WMSDs), according to WHO [2]. WMSDs are partly preventable given the association with work-related risk factors. With regard to physical ergonomic risk factors such as force exertion, demanding posture or repetitive movement, recent studies found that occupational exposure is highly prevalent and there is evidence that the burden of MSDs attributed to that exposure is substantial [6, 7]. For several prevalent musculoskeletal disorders, threshold limits are formulated for work-related risk factors. Examples are carpal tunnel syndrome [8], lateral epicondylitis [9, 10], specific shoulder disorders [11], hip and knee osteoarthritis [12, 13] and lumbosacral radiculopathy syndrome [14].

To eliminate or minimize the work-related risk factors for MSD, primary prevention is widely advocated. A framework of six steps was proposed in 2017 for setting up such prevention [15]. The first three steps include identifying the incidence and severity of the condition, determining the risk factors that may be involved and the mechanisms that may cause a MSD. In the fourth step, based on the knowledge of previous steps, an intervention is developed. Steps five and six concern the evaluation and implementation of the assumed effective intervention.

When developing an intervention, different interventions can be distinguished [16, 17]. There are interventions to improve organisational aspects of work, aimed for example at the task content, collaboration, support, work pace and planning. There are also technical interventions with the focus on for instance the work environment, working height, tools and equipment. And there are interventions regarding the behaviour of workers, addressing working practice, education and training.

To emphasize the context of work, the term Individual Working Practice (IWP) is used to describe the behaviour of workers in this study. IWP covers both short term individual behaviour that influences work-related physical ergonomic risk factors, like posture and working speed, and skills acquired over time that influence these risk factors, like motoric skills and professional competence.

These different types of interventions can be combined in an implementation project. Where possible, the hierarchy of risk management should be taken into account, i.e. organisational and technical measures are preferred over interventions aimed at behaviour [18].

Although IWP takes last place in the hierarchy of risk management, in the context of preventing WMSDs it still is a key topic for various reasons. First, a technical or organisational improvement may not be possible or not immediately available. A behavioural change, if effective, is then a logical next step. Second, the success of a technical or organisational improvement can depend on the behaviour and compliance of the employee. For instance, lifting equipment only has an effect if it is used in daily practice, halving the weight of the cement bag is only an improvement if the mason lifts one bag instead of two. Third, improving personal behaviour is the only topic that can be intervened on during the course of a vocational training; an improvement in the later work organisation or the work environment is obviously not part of the curriculum. Fourth, when WMSDs are treated in curative care, the health practitioner usually has no direct control over the work environment or work organisation. However, behaviour in daily work practice can be influenced and is therefore a feasible starting point for this guidance.

There are numerous articles in the medical literature describing interventions that include an aspect of IWP. For example, if the intervention comprises of advice on posture, working technique or work variation. It is striking to note that, as far as we are aware, in the context of prevention of WMSDs, no framework exists for the categorisation of interventions for IWP. To take a first step towards the development of such a framework, this study answers the question: which categories of interventions for Individual Working Practice (IWP) can be distinguished to reduce exposure to physical ergonomic risk factors in order to prevent WMSDs?

## Method

To answer the research question, a scoping review as designed by Arksey [19], later supplemented by Levac [20], and in line with the PRISMA-Scoping Reviews extension [21] and the JBI reviewer’s manual [22], was performed. The prescribed steps, with the exception for ‘the consultation step’, have been completed. The subsequent steps are: developing search strategy, identifying relevant studies, data charting, collation and discussion.

### Search strategy

To develop a search strategy, a non-systematic search was first performed via PubMed. The aim was to find a select group of about twenty papers with IWP as their subject. In joint consultation, agreement was reached on twenty-three papers (PK, BV, BW). By analysing these papers in a mutual consultation (PK, BV, BW, JD), the clinical librarian JD distilled search terms for an extensive systematic search. The databases Ovid Medline and Ovid Embase were chosen because these databases represent the majority of the scientific literature on prevention of work-related musculoskeletal disorders. To cover interventions with a psychological component, APA PsycInfo has been added and Web of Science to cover conference proceedings. There was no restriction regarding the years of publication.

The set of preselected twenty-three papers was used to assess whether the developed search strategy has found all these papers. If not, the search was modified using an iterative process. The final search strategy is outlined in Additional file 1: Appendix B.

### Identify relevant studies and study selection

Inclusion criteria were: 1. Abstract available (exclusion label: ‘No abstract); 2. English language (exclusion label: ‘Foreign Language’); 3. Full text available and primary study, no review (exclusion label: ‘Wrong publication type/Wrong study design‘); 4. Papers must relate to work, workers or working practice (exclusion label: ‘No work’); 5. Papers must relate to physical ergonomic work-related risk factors or physical workload (exclusion label: ‘No Physical load’); 6. Papers must relate to IWP, and should describe an intervention or measure aimed to reduce exposure to one or more physical ergonomic risk factors that can be influenced by the worker (exclusion label: ‘No IWP’); 7. Papers should describe the effect of the intervention or measure in terms of exposure to the physical ergonomic risk factors (exclusion label: ‘Wrong outcome’).

No quality assessment of the studies has purposefully been performed. The aim was to trace as many types of IWP interventions as possible. By performing a selection on the quality of the research, a selection bias could be introduced, for example on more simply described IWP interventions or IWP interventions that have only been described in non-randomised observational studies without a control group.

BW performed the first inclusion of relevant papers by scanning title and abstract. In this phase there was a meeting every 2 weeks with PK and BW in which the studies that potentially complied with the inclusion criteria were discussed until agreement was reached. Thereafter, PK and BV jointly scanned 15 studies to evaluate whether the right papers had been included and to calibrate their assessment. Subsequently, PK and BV then independently reassessed each half of all studies that were included in the first global scan. The results of this reassessment was discussed in mutual consultation (PK,BV,BW) to identify papers eligible for full text reviewing and data charting. In case of doubt, the study was included in the full-text screening.

### Data charting

A data extraction sheet was designed to collect information from the selected studies. BW performed the data-charting of five articles according to this chosen design and this was discussed (BW, PK and BV). The following data were extracted: 1st author – Title Year of publication – Country – Study design – The aim of the intervention/measure– IWP intervention topics – IWP intervention outcome measured – Results of the IWP intervention on the outcome –Number of people (workers) involved – Age – Sex - Kind of work – Remarks.

Data extraction of the selected studies was performed by BW as described in the studies and these data were checked by BV and PK.

### Collation, summarising and reporting

All IWP intervention topics collected in data charting were merged by BW into an overview in Excel. Next the following steps were taken. First, similar topics were combined. Then an inductive approach was used to code the extracted data by asking: what has the worker to do, change or develop to reduce the exposure to a physical ergonomic risk factor? For example, the topic ‘correct monitor position’ leads to the code: ‘adjusting workplace’, because that is what the worker has to do. Other topics like ‘mouse position’, ‘right position of the bed’, ‘the workstation lay-out’ also fit in this code. Another example is ‘patient-handling techniques’ leading to the code: ‘motoric skill’, because the worker has to develop or to apply that skill. Topics like ‘the correct lifting posture’, ‘the correct hand position’ fit also in this code. If a topic did not fit into an existing code, a new code was named. In the distinction between codes, the process to achieve the change was an important factor to base the decision on. For example, it is a different process to make a change within an activity, i.e. to change working from left to right hand, than to change a task schedule over a day; the first can be seen as an example of variation in working technique and the latter as an example of task content & task organisation. During mutual consultations between BW, BV and PK, all topics were discussed and coded. Coding discrepancies were discussed until 100% agreement was achieved. Finally these codes were defined as the categories of interventions for IWP according which exposure to physical ergonomic risk factors can be reduced in order to prevent WMSDs.

## Results

### General

**Fig. 1:**
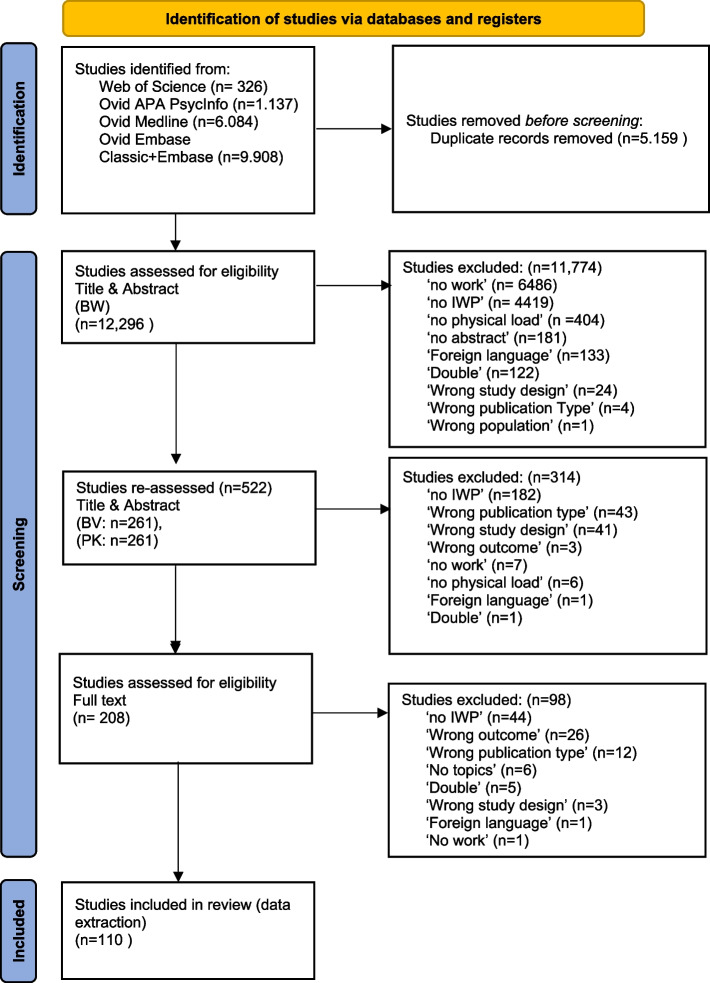
Flowchart of selection of papers

The systematic search until July 2021 generated 17.455 articles. Most articles were found in Ovid Medline (> 6000) and Ovid Embase (> 9000). There was an overlap of more than 5000 articles. The first screening on title and abstract involved 12,296 articles. After this screening, 522 studies remained. Most studies were excluded because of the No Work or No IWP label. Of these, after a second screening by PK (261) and BV (261), 314 studies were eligible for full text review and data charting. In that process another 204 studies were excluded, most of them because it turned out it wasn’t about IWP. Ultimately 110 studies fulfilled the inclusion criteria, 51 from PK and 59 from BV. The flowchart of the selection process is depicted in Fig. 1.

**Fig. 2:**
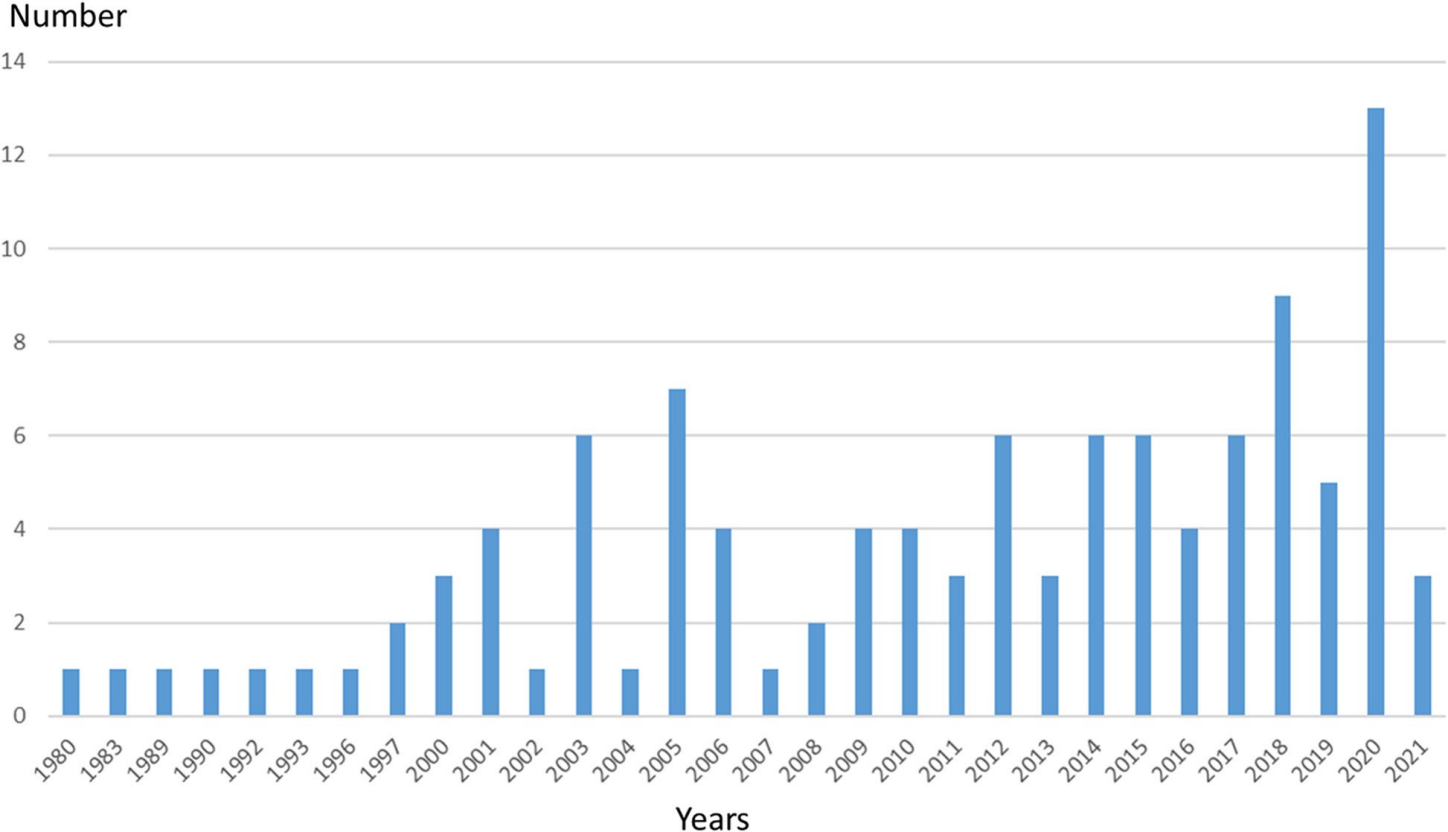
Overview of included studies by year of publication

The included articles described interventions or measures aimed at a wide variety of work activities. Office work is the main part (44), nursing is second (32), and other studies are performed in construction work (14), assembly work (14), manual material handling (6), working in the meat industry (6), driving (3), dentistry (3), teaching (3), kitchen work (3), cleaning (2), and more. In the distribution over the years, we see a gradual increase of included studies in the period from the start in 1980 up to and including 2021 (Fig. 2 ). With the exception of Africa, the studies are performed in the following continents: North America (46), Asia (31), Europe (25), Oceania (7) and South America.

### Topics

The 110 included studies described in total 819 intervention topics concerning IWP. For example, a study on prevention among healthcare workers yielded 15 intervention topics, such as lifting technique, patient assessment and using smooth controlled movements [23]. A study about an intervention in computer workers yielded two intervention topics, namely workplace adjustments and workplace exercises [24] and a study of an educational program among school teachers yielded twelve intervention topics, such as doing breaks, doing exercises and adjusting body joint angles. All these topics are described in the Additional file 1: Appendix C including the references to the studies concerned.

### Categorisation

The topics are coded according to the question: what has the worker to do, change or develop to reduce the exposure to a physical ergonomic risk factor? In total 160 topics were coded as Workplace adjustments. For example topics like chair adjustments, correction of the mouse position or the position of the bed. In total 59 topics, like varying work posture, alternate between both hands and incorporate minibreaks are coded as Variation. This is variation within a work-related activity. Exercising is a category in which 56 topics were included that have to do with a form of physical training aimed at fitness, strength and relaxation exercises. Use of aids, including 58 topics, is about the use of supporting tools, like for example lifting equipment. Professional skills, with 53 topics, is the category that contains specific skills strongly related to the job and where proper application of these skills can reduce exposure to physical ergonomic risk factors. Examples are a specific cutting technique of the deboner in the meat industry or the dexterity in the care of patients. The category Professional manners, with 86 topics in it, may appear similar to the previous category of Professional skills. However, in contrast to Professional skills, Professional manners is about professional behavior, such as working together, following rules and making preparations. Task content and task organisation (15 topics) is the category that contains topics related to planning and coordination of activities or alternating between activities within the work. For example time-management, task modification or pacing during the workday. The most frequently described intervention topics were coded as Motoric skills, namely with 323 times. This category includes topics related to specifically trained movements to perform the work with less exposure to physical ergonomic factors, such as using less extreme body joint postures while performing an activity or preventing a twisted back when picking up loads.

In summary, if a different IWP strategy is needed to reduce exposure to a physical ergonomic risk factor, another category has been formulated based on the described topics. Adjusting a workplace differs from training a motoric skill. Using a tool differs from adjusting the order in which work activities are performed. The distinction of the eight categories provides the opportunity to develop specific knowledge on the effectiveness of the categories on the prevention of work-related MSDS or a targeted approach for implementation. The definition of these categories are thus a first step towards a framework for interventions for IWP to prevent work-related musculoskeletal disorders.

The coding of the 819 intervention topics resulted eventually in eight categories of interventions for IWP. Table 1 gives an overview of these 8 categories, including some examples and the references to the related studies. The table in Additional file 1: Appendix C shows all intervention topics per category including the references.

**Fig. 3:**
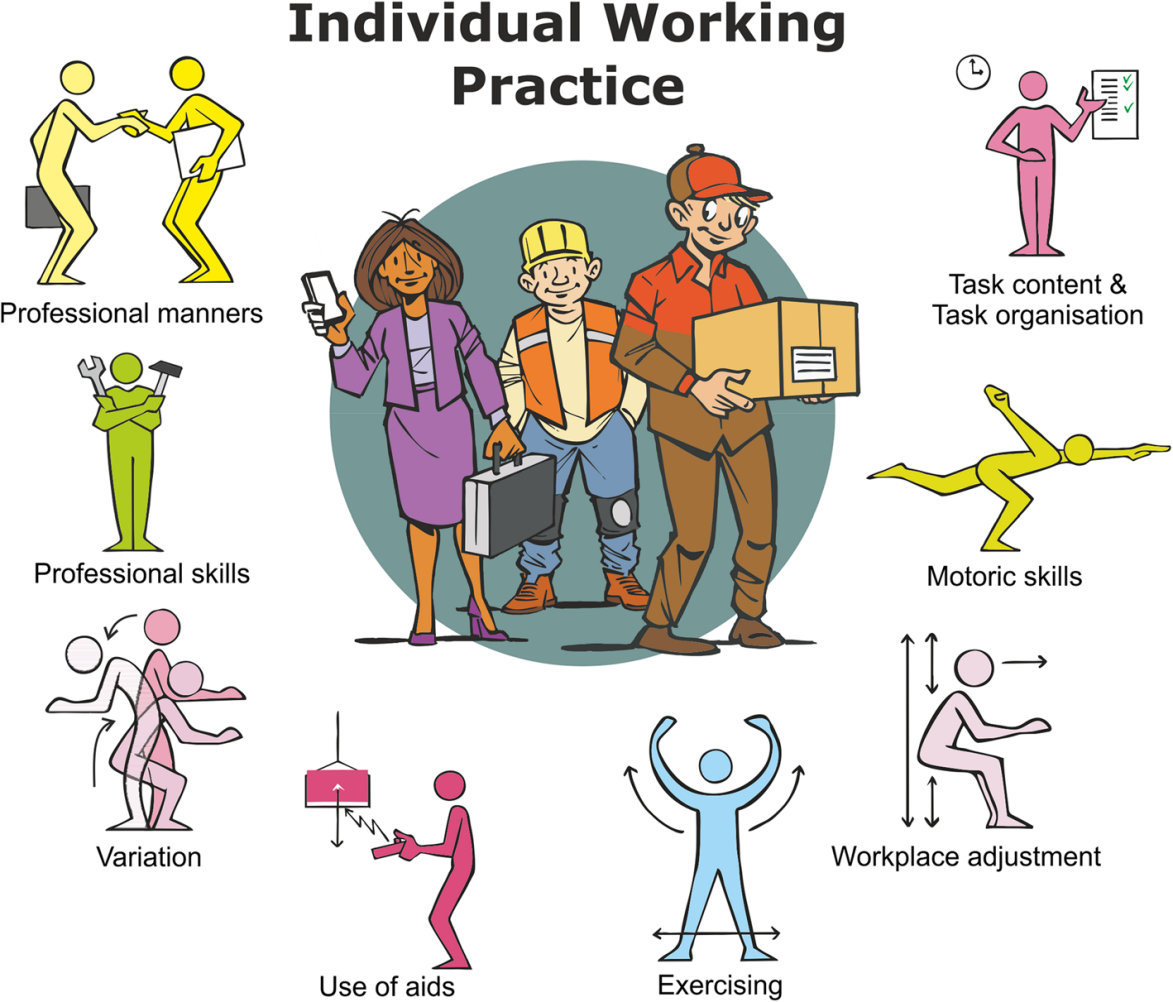
Eight categories of interventions for Individual Working Practice (IWP)

In Fig. 3 a graphical representation of the categories of interventions for IWP is displayed. In doing so, a symbolic representation of each category was sought that fits the definition. These symbols can be of added value in applying and communicating these IWP interventions with workers.

**Table 1:** Categories of interventions for Individual Working Practice and some examples, included references

Categories		Studies containing intervention topics belonging to this category
1. Workplace adjustments: the worker adjusts the working environment. (160 topics) Examples: • Proper positioning chair- desk-monitor- keyboard-mouse-footstools-printer- scanner- telephone- frequently used objects- bed • Adjustment work tool and office equipment • Lowering the storage position of heavy equipment		[25]; [26]; [27]; [28]; [29]; [30]; [31]; [32]; [33]; [34]; [35]; [36]; [24]; [37]; [38]; [39]; [40]; [41]; [42]; [43]; [44]; [45]; [46]; [47]; [48]; [49]; [50]; [51]; [52]; [53]; [54]; [55]; [56]; [57]; [58]; [59]; [60]; [61]; [62]; [63]; [64]; [65]; [66]; [67]; [68]; [69]; [70]; [71].
2. Variation: the worker varies in the execution of the work activities. (59 topics) Examples: • Varying work posture, positioning in work • Alternation between hands, pressing finger etc. • Taking (micro) rest-breaks, using work-rest schedules		[25]; [72]; [73]; [28]; [29]; [30]; [74] ; [75]; [76]; [36]; [77]; [39]; [41]; [78]; [79]; [80]; [45]; [46, 74]; [50]; [51]; [52]; [53]; [81]; [82]; [83]; [60]; [61]; [63]; [64]; [65]; [66]; [68]; [69]; [84]; [70]; [71].
3. Exercising: the worker executes a form of physical training to prepare for work or to recover from the effects of work. (56 topics). Examples: • Stretching, strengthening or relaxing exercises • Warm-up routines • Posture correction exercises		[26]; [27, 85]; [28]; [31]; [34]; [23]; [76]; [24]; [86]; [77]; [38]; [39]; [41]; [87]; [46]; [47]; [52]; [53]; [62]; [64]; [88]; [65]; [66]; [68]; [69]; [70]; [89]; [90].
4. Use of aids: the worker chooses to use assistive tools. (58 topics) Examples: • Ceiling lift usage • Using transfer equipment • Using of document holder, headset phone, optical tool		[91]; [92]; [73]; [31]; [93] ; [32]; [33]; [23]; [94]; [35]; [76]; [86]; [37] ; [38]; [39]; [49]; [51]; [52]; [53]; [81]; [58]; [59]; [60]; [62]; [65]; [66]; [69]; [95]; [96]; [70].
5. Professional skill: the worker applies specific job related know-how and dexterity that facilitates the execution of the work. (53 topics) Examples: • Specific professional technics for deboning: grip forces, cutting moments, position of the piece of meat etc. • Specific professional working technique in nursing (f.i. replace clothing). • Specific professional technics by drivers: route selection, driving style, driving speed		[73]; [97]; [76]; [77]; [42]; [43]; [87]; [98]; [79]; [80]; [46]; [48]; [49]; [51]; [56]; [59]; [82]; [63]; [88]; [66]; [99]; [70]; [100].
6. Professional manners: the worker applies specific job related behaviour that facilitates the execution of the work. (86 topics) Examples: • Ask help from colleagues • Patient assessment before handling • Compliance with recommendations (f.i. no lift policy)		[73]; [29]; [31]; [101]; [23]; [97]; [76]; [86]; [77]; [43]; [79]; [45]; [49]; [51]; [53]; [59]; [102]; [82]; [103]; [62]; [84]; [104] ; [95]; [70];
7. Task content and task organisation: the worker changes the content of a specific part of the work or the way these parts are scheduled to get more variety in the execution of work. (15 topics) Examples: • Improve work organisation • Managing the pace of one’s own work • Time-management		[28]; [74]; [39]; [41]; [42]; [51]; [53]; [57]; [82]; [67]; [68]; [70].
8. Motoric skill: the worker applies specific trained movements to perform the work with less physical strain. (323 topics) Examples: • More relaxed (neutral) positions in joints • Patient-handling techniques • Accurate acceleration-velocity-timing in movement		[25]; [92]; [105]; [73]; [27]; [28]; [106]; [29]; [107] ; [30]; [31] ; [93]; [101]; [108]; [34]; [23]; [94]; [35]; [75]; [76]; [86]; [77]; [109]; [110]; [37]; [111]; [112]; [39]; [113]; [40]; [41]; [42]; [43]; [44]; [87]; [98]; [114]; [79]; [80]; [45]; [115]; [116]; [46]; [47]; [48]; [49]; [117]; [50]; [51]; [52]; [53]; [54]; [55]; [56]; [58]; [118]; [102]; [82]; [103]; [119]; [83]; [60]; [120]; [121]; [62]; [122]; [123]; [64]; [124]; [88]; [65]; [66]; [67]; [68]; [125]; [69]; [104]; [95]; [126]; [96]; [127] ; [70]; [100]; [89]; [128] ; [129]; [130]; [90].; [71].

## Discussion

Based on this scoping review, a first step towards a conceptual framework for interventions for IWP is made to prevent WMSDs due to physical ergonomic risk factors. Eight categories of interventions for IWP are distinguished: Workplace adjustment, Variation, Exercising, Use of aids, Professional skills, Professional manners, Task content & task organisation and Motoric skills.

### Relevance

The categorisation of interventions can be helpful in designing and in evaluating the effectiveness of these interventions For example, the distinction made between organisational, technical and behavioural interventions in prevention of WMSDs makes it possible to prioritise one approach above the other. The same kind of prioritisation seems also possible for the eight categories of interventions for IWP. For example: the ratio between effort and effect of an intervention probably differs between stimulating a worker to change the workplace versus training a motor skill in order to prevent WMSDs. Besides prioritisation of interventions the categorisations also offers the opportunity to apply the right approach for successful implementation. Regarding the former example, stimulating a worker to change the workplace versus training motoric skills requires different expertise and training or teaching skills. Adding the right approaches for each category makes the framework probably even more useful for theory and practice.

On an individual level, a framework might facilitate communication to prevent WMSDs among workers, thereby increasing shared understanding, and sharing power and responsibility – two of the four important domains in consultations [131]. At a community level, a framework is regarded as an essential prerequisite for advancing the translation of science on prevention into practice [132].

These developments can support occupational health professionals and workers alike in designing, evaluating and implementing effective IWP interventions and thus reducing exposure to physical ergonomic risk factors for WMSDs. A framework could also encourage communication between researchers, practitioners, employees and employers, strengthening the field of IWP.

### Ranking

Interventions for IWP to reduce exposure to work-related physical ergonomic risk factors might overlap with interventions based on technical and organisational measures. For example, a workplace adjustment can also be an assignment for the company and a strictly imposed work pace might negatively influence the IWP. Above all, a focus on the IWP should not divert attention from the other two types of ergonomic interventions that have a higher priority in the hierarchy of prevention. In addition IWP should of course not be a stepping stone to blame the worker for harmful exposure to physical ergonomic risk factors. Moreover, improvements in the IWP are often the result of a learning process and therefore dependent on the effectiveness of that process and on the workers responsiveness. Eliminating, if possible, the source of exposure to physical ergonomic risk factors is always the best course of action.

### Strength and limitations

A strength of the present review is that the categorisation of interventions for IWP is based on an extensive literature search in four databases using a validation set of preselected papers. A second strength is that because of the inclusion criterion ‘Outcome measured must relate to IWP intervention topics’, all topics were of relevance for practice and therefore do justice to the P of IWP. However, an important limitation is that using only literature does not guarantee that all relevant topics and categories have been identified. Moreover, the coding was done by only three Dutch experts in the field of WMSD prevention. It is therefore necessary to test the categorisation and the vocabulary used in a broad consultation of international experts in this field. This international consultation is also the final step in the performance of a scoping review [19, 20] and therefore it will be our next step to assess if the presented eight categories are seen as a valid representation of the current literature.

Another limitation might be that the first selection of the more than 12.000 papers was only performed by the first author with a random calibration with the other two researchers. This might have resulted in selection bias. However, the ultimately included studies have been approved by all three researchers. Moreover, we presume that the selection bias is probably small due to the broad search and the large number of papers that were included. If papers have been missed than the more than 800 extracted topics from the selected papers still appear a reliable source for the coding of the topics in the eventual eight categories of interventions for IWP. At least 15 studies were found per category, so that the chance is considered small that an entire category was missed due to this design of the selection procedure. In addition, the international consultation of experts will further minimize this effect.

We purposefully selected only papers describing interventions or measures aimed at reducing exposure to physical ergonomic risk factors or physical workload. This is a limitation because interventions exclusively aimed at psychosocial risk factors are excluded. The reason for this choice is that the psychosocial factors partly belong to the organisational domain, such as work atmosphere and collegiality. Moreover, in the context of IWP to prevent WMSDs it is unlikely that only psychosocial risk factors are a direct cause for the development of WMSDs, without a physical ergonomic risk factor being involved. Nevertheless, this may be considered an omission, which needs further study in the future.

Distinguishing between categories and assigning an intervention to a category might be arbitrary. Sometimes the distinction is clear, like for example the difference between a motor skill and a workplace adjustment. Sometimes the distinction is less clear, for example the difference between a professional skill and a professional manner. Sharpening a knife was categorized as a professional skill and compliance with rules was categorized as a professional manner. However, in case of verbally guiding a patient while transferring him or her from chair to bed, this distinction might be more diffuse. The choice to verbally guide the patient was eventually seen as a professional manner, but the verbally guiding was seen as a professional skill. Taking a mini-break during an activity was categorised as variation and how long this mini-break should last as an example of task content and organisation. Of course other experts might think differently about these choices made. However the main contribution of the present paper is the definition of these eight categories. Here too, an international expert consultation might further improve and/or strengthen the IWP intervention framework.

### Improving IWP

This paper presents a first step into the development of an IWP framework. Once the eight categories are seen as describing distinct IWP interventions, meta-analyses can be performed to assess the effect of each category on reduction of exposure to physical ergonomic risk factors and on the actual prevention of work-related MSDs.

Furthermore, it is likely that in the practical implementation of the interventions, different categories also require different approaches. For example, encouraging and guiding a worker in changing a motor skill requires a different approach than supporting an improvement in professional manners. A suitable strategy to teach or train workers regarding the eight categories of interventions in IWP, might be adapted from the approaches to improve performance in sports. In sports four different approaches are distinguished, because improve personal behavior requires different learning processes [133–135]. The technical approach concerns unconsciously, automated skills. The physiological approach concerns the physical capabilities of the person, such as agility, strength, endurance, general health. The psychological approach addresses the psychological aspects of behaviour, like motivation, attention, and stress resistance. The tactical approach addresses consciously made choices or decisions made for best performance.

Regarding the eight categories of interventions in IWP, the technical approach seems relevant to address Professional skills and Motoric skills. For Workplace adjustments, Variation, Use of aids, Task content and task organisation a tactical approach seems most appropriate and for Professional manners a psychological approach. Finally, a physiological approach seems most appropriate to address Exercising (Figure A1 in  Additional file 1: Appendix A).

## Conclusion

A first step towards a conceptual framework for interventions for IWP is made by defining eight categories of interventions, based on the scientific literature, like workplace adjustment, motor skills and variation. These categories can be used as a starting point for developing and evaluating the effectiveness of these worker-oriented interventions to prevent WMSDs.

## Supplementary Information

**Additional file 1:** **Appendix A**. Frameworkof interventions for Individual Working Practice (IWP) included 4 approaches for improvement. **Figure A1**. Eight categories of interventions for Individual Working Practice (IWP) included 4 approaches for improvement as mentioned in the Discussion chapter. **AppendixB**. SEARCH-STRATEGY. **Appendix C**. Topic List Per Category.

## Data Availability

The datasets generated and analysed during the current study are available from l.c.vandewijdeven@amsterdamumc.nl upon reasonable request.

## Abbreviations

UMC: University Medical Centre
WMSDs: Work-related musculoskeletal disorders
IWP: Individual Working Practice
BW: Bert van de Wijdeven
BV: Bart Visser
PK: Paul Kuijer
JD: Joost Daams
