# Supplementary material for: A first step towards a framework for interventions for individual working practice to prevent work-related musculoskeletal disorders: a scoping review
Source: BMC Musculoskelet Disord. 2023 Feb 1;24:87. doi: 10.1186/s12891-023-06155-w (PMC9890723; doi:10.1186/s12891-023-06155-w)
Supplement: Supplementary file 1 — Additional file 1: Appendix A. Frameworkof interventions for Individual Working Practice (IWP) included 4 approaches for improvement. Figure A1. Eight categories of interventions for Individual Working Practice (IWP) included 4 approaches for improvement as mentioned in the Discussion chapter. AppendixB. SEARCH-STRATEGY. Appendix C. Topic List Per Category. [file 12891_2023_6155_MOESM1_ESM.docx]

**A first step towards a framework for interventions for Individual Working Practice to prevent work-related musculoskeletal disorders: a scoping review**

**APPENDICES**

**APPENDIX A: Framework of interventions for Individual Working Practice (IWP) included 4 approaches for improvement.**


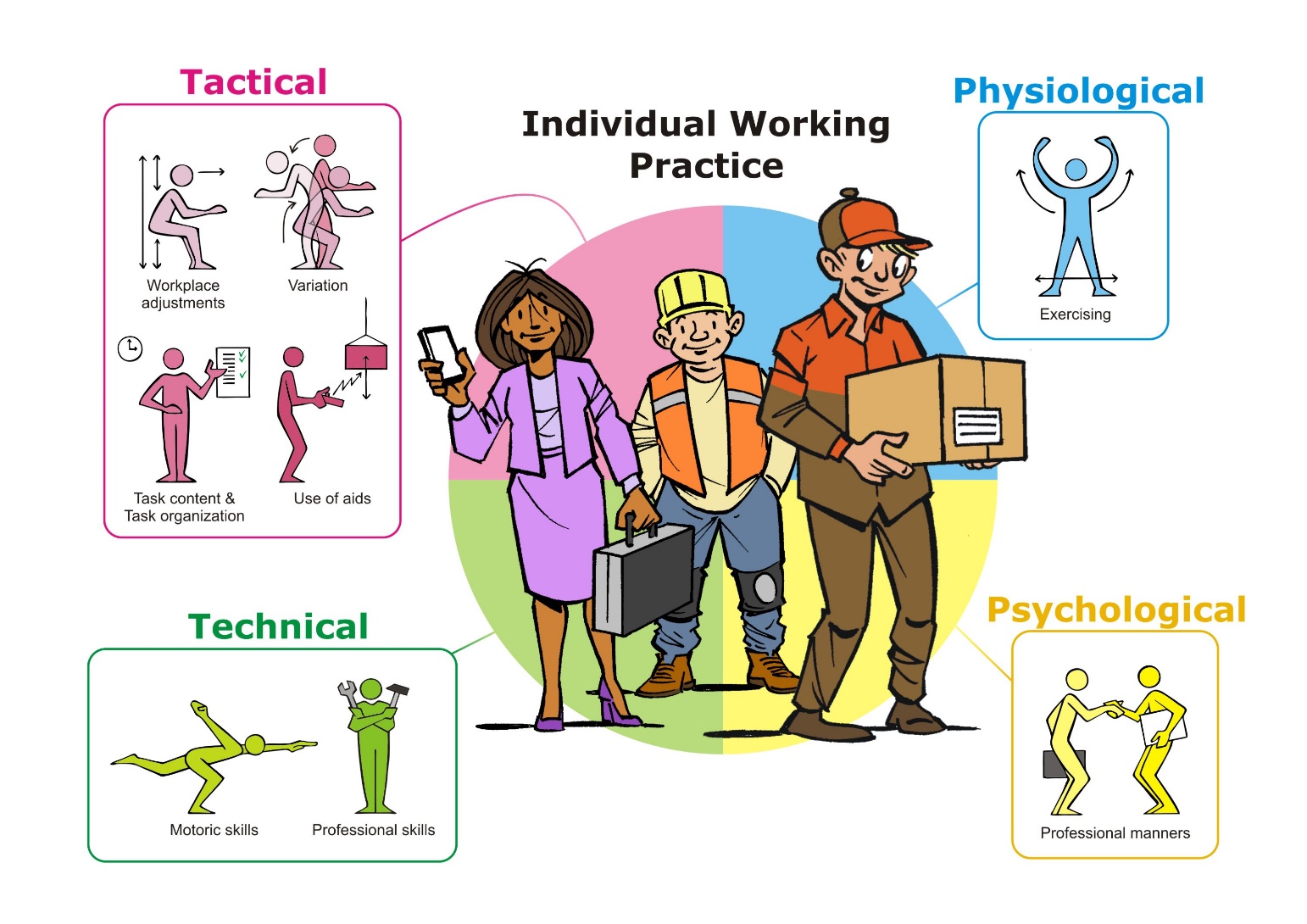


Figure A1: Eight categories of interventions for Individual Working Practice (IWP) included 4 approaches for improvement as mentioned in the Discussion chapter.

**APPENDIX B: SEARCH-STRATEGY**

|  | **Ovid MEDLINE(R) ALL <1946 to July 28, 2021> Search date: 29 July 2021** |  |
| --- | --- | --- |
| **#** | **Searches** | **Results** |
| 1 | ergonomics/mt | 1335 |
| 2 | "Moving and Lifting Patients"/ | 661 |
| 3 | (movement control or working method? or workstyle? or ergomotor or motor control or motor variability or job control).ab,kf,ti. | 17792 |
| 4 | ((agile or execution) and (task? or technique? or work)).ab,kf,ti. | 10842 |
| 5 | ((skill? or technique?) adj3 (handl* or lift* or lowering)).ab,kf,ti. | 2889 |
| 6 | (worker? adj3 competence).ab,kf,ti. | 108 |
| 7 | (skill? adj3 development).ab,kf,ti. | 6712 |
| 8 | health education/ and ergonomics/ and (occupational diseases/ or occupational health/) | 50 |
| 9 | ((coaching or advice or instruction? or programme? or teaching or educati* or didact* or autodidact* or training) and (adjust* workstations or manual handling or ergonomics) and prevention).ab,kf,ti. | 180 |
| 10 | Health Knowledge, Attitudes, Practice/ and (occupational diseases/ or occupational health/) | 1279 |
| 11 | (work* adj3 practice).ab,kf,ti. | 8424 |
| 12 | (worker? adj2 (belie* or conviction?)).ab,kf,ti. | 244 |
| 13 | work pace.ab,kf,ti. | 231 |
| 14 | (additional work break? or relaxation work break?).ab,kf,ti. | 1 |
| 15 | (job coaching and prevention).ab,kf,ti. | 2 |
| 16 | (behavio?r* adj2 chang*).ab,kf,ti. | 54752 |
| 17 | (risk and (movement? or ergonomic* or posture?)).ab,kf,ti. | 22913 |
| 18 | pacing.ab,kf,ti. | 33830 |
| 19 | exp Occupations/ or Workload/ or exp Work/ or Workplace/ or exp Occupational Diseases/ or Rehabilitation, Vocational/ or Occupational Health/ or Sick Leave/ or Absenteeism/ or workers' compensation/ or exp Employment/ or exp Occupational Exposure/ or disability evaluation/ | 413542 |
| 20 | (worka* or worke* or workg* or worki* or workl* or workp* or work capacity or work disabilit* or work abilit* or at work or work exposure or work related or workers or job* or employee or staff or personnel or occupation or occupations or occupational or outdoor work* or day shift* or night shift* or shift work* or vocational rehabilitation or sick leave or absenteeism or sickness absen* or absente* or presente* or "return to work" or vocational reintegration or employment or unemployed or unemployment or work status or industries or industrial sector or repetitive work or wet work or (disability adj2 (assess* or evaluation))).ab,kf,ti. | 2122105 |
| 21 | 19 or 20 [work filter] | 2315619 |
| 22 | (16 or 17 or 18) and 21 | 16499 |
| 23 | or/1-15,22 [individual work approach] | 65659 |
| 24 | (musculoskeletal or foot or feet or ankle? or knee? or hip? or coxa or limb? or low back or back pain or shoulder? or spine or spinal or elbow? or wrist? or hand? or neck).mp. | 1961439 |
| 25 | (prevention or risk or epidemiology or etiolog*).mp. | 6971322 |
| 26 | and/23-25 | 6084 |
|  |  |  |
|  |  |  |
|  | **Ovid Embase <1947 to 2021 July 28>  Search date: 29 July 2021** |  |
| **#** | **Searches** | **Results** |
| 1 | *ergonomics/ | 5663 |
| 2 | (movement control or working method? or workstyle? or ergomotor or motor control or motor variability or job control).ab,kw,ti. | 24308 |
| 3 | ((agile or execution) and (task? or technique? or work)).ab,kw,ti. | 13693 |
| 4 | ((skill? or technique?) adj3 (handl* or lift* or lowering)).ab,kw,ti. | 3762 |
| 5 | (worker? adj3 competence).ab,kw,ti. | 129 |
| 6 | (skill? adj3 development).ab,kw,ti. | 8721 |
| 7 | health education/ and ergonomics/ and (occupational disease/ or occupational health/) | 43 |
| 8 | ((coaching or advice or instruction? or programme? or teaching or educati* or didact* or autodidact* or training) and (adjust* workstations or manual handling or ergonomics) and prevention).ab,kw,ti. | 278 |
| 9 | attitude to health/ and (occupational disease/ or occupational health/) | 1176 |
| 10 | (work* adj3 practice).ab,kw,ti. | 10918 |
| 11 | (worker? adj2 (belie* or conviction?)).ab,kw,ti. | 327 |
| 12 | work pace.ab,kw,ti. | 273 |
| 13 | (additional work break? or relaxation work break?).ab,kw,ti. | 1 |
| 14 | (job coaching and prevention).ab,kw,ti. | 1 |
| 15 | (behavio?r* adj2 chang*).ab,kw,ti. | 70365 |
| 16 | (risk and (movement? or ergonomic* or posture?)).ab,kw,ti. | 34393 |
| 17 | pacing.ab,kw,ti. | 52511 |
| 18 | exp Occupation/ or Workload/ or exp Work/ or exp occupational health/ or exp Occupational Disease/ or medical Leave/ or workman compensation/ or exp Occupational Exposure/ | 946621 |
| 19 | (worka* or worke* or workg* or worki* or workl* or workp* or work capacity or work disabilit* or work abilit* or at work or work exposure or work related or workers or job* or employee or staff or personnel or occupation or occupations or occupational or outdoor work* or day shift* or night shift* or shift work* or vocational rehabilitation or sick leave or absenteeism or sickness absen* or absente* or presente* or "return to work" or vocational reintegration or employment or unemployed or unemployment or work status or industries or industrial sector or repetitive work or wet work or (disability adj2 (assess* or evaluation))).ab,kw,ti. | 3456276 |
| 20 | 18 or 19 [work filter] | 3950123 |
| 21 | (15 or 16 or 17) and 20 | 31504 |
| 22 | or/1-15,21 [individual work approach] | 154821 |
| 23 | (musculoskeletal or foot or feet or ankle? or knee? or hip? or coxa or limb? or low back or back pain or shoulder? or spine or spinal or elbow? or wrist? or hand? or neck).mp. | 2874621 |
| 24 | (prevention or risk or epidemiology or etiolog*).mp. | 8784858 |
| 25 | and/22-24 | 9908 |
|  |  |  |
|  |  |  |
|  | **Ovid APA PsycInfo <1806 to July Week 3 2021> Search date: 29 July 2021** |  |
| **#** | **Searches** | **Results** |
| 1 | exp human factors engineering/ | 9044 |
| 2 | (movement control or working method? or workstyle? or ergomotor or motor control or motor variability or job control).ab,id,ti. | 10404 |
| 3 | ((agile or execution) and (task? or technique? or work)).ab,id,ti. | 6550 |
| 4 | ((skill? or technique?) adj3 (handl* or lift* or lowering)).ab,id,ti. | 653 |
| 5 | (worker? adj3 competence).ab,id,ti. | 157 |
| 6 | (skill? adj3 development).ab,id,ti. | 11232 |
| 7 | (health promotion or health education).mp. and exp human factors engineering/ and exp occupational health/ | 6 |
| 8 | ((coaching or advice or instruction? or programme? or teaching or educati* or didact* or autodidact* or training) and (adjust* workstations or manual handling or ergonomics) and prevention).ab,id,ti. | 64 |
| 9 | health attitudes/ and exp occupational health/ | 17 |
| 10 | (work* adj3 practice).ab,id,ti. | 13449 |
| 11 | (worker? adj2 (belie* or conviction?)).ab,id,ti. | 263 |
| 12 | work pace.ab,id,ti. | 133 |
| 13 | (additional work break? or relaxation work break?).ab,id,ti. | 1 |
| 14 | (job coaching and prevention).ab,id,ti. | 1 |
| 15 | (behavio?r* adj2 chang*).ab,id,ti. | 39865 |
| 16 | (risk and (movement? or ergonomic* or posture?)).ab,id,ti. | 6117 |
| 17 | pacing.ab,id,ti. | 2116 |
| 18 | (work* or occupation* or labour or vocational or employment).hw. | 178909 |
| 19 | occupation*.cc. | 29496 |
| 20 | (worka* or worke* or workg* or worki* or workl* or workp* or work capacity or work disabilit* or work abilit* or at work or work exposure or work related or workers or job* or employee or staff or personnel or occupation or occupations or occupational or outdoor work* or day shift* or night shift* or shift work* or vocational rehabilitation or sick leave or absenteeism or sickness absen* or absente* or presente* or "return to work" or vocational reintegration or employment or unemployed or unemployment or work status or industries or industrial sector or repetitive work or wet work or (disability adj2 (assess* or evaluation))).ab,id,ti. | 901716 |
| 21 | or/18-20 [work filter] | 949893 |
| 22 | (15 or 16 or 17) and 21 | 8758 |
| 23 | or/1-14,22 [individual work approach] | 59322 |
| 24 | (musculoskeletal or foot or feet or ankle? or knee? or hip? or coxa or limb? or low back or back pain or shoulder? or spine or spinal or elbow? or wrist? or hand? or neck).mp. | 186403 |
| 25 | (prevention or risk or epidemiology or etiolog*).mp. | 587920 |
| 26 | and/23-25 | 1137 |
|  |  |  |
|  |  |  |
|  | **Web of Science: conference proceedings Search date: 29 July 2021** |  |
| **#** | **Searches** | **Results** |
| 1 | TS=(movement control or working method? or workstyle? or ergomotor or motor control or motor variability or job control) | 762861 |
| 2 | TS=((agile or execution) and (task? or technique? or work)) | 30737 |
| 3 | TS=((skill? or technique?) NEAR/2 (handl* or lift* or lowering)) | 5551 |
| 4 | TS=(worker? NEAR/2 competence) | 181 |
| 5 | TS=(skill? NEAR/2 development) | 8942 |
| 6 | TS=(((coaching or advice or instruction? or programme? or teaching or educati* or didact* or autodidact* or training) AND (adjust* workstations or manual handling or ergonomics)) and prevention) | 321 |
| 7 | TS=(work* NEAR/2 practice) | 27225 |
| 8 | TS=(worker? NEAR/1 (belie* or conviction?)) | 328 |
| 9 | TS=(work pace) | 7730 |
| 10 | TS=(additional work break? or relaxation work break?) | 412 |
| 11 | TS=(job coaching and prevention) | 19 |
| 12 | #1 OR #2 OR #3 OR #4 OR #5 OR #6 OR #7 OR #8 OR #9 OR #10 OR #11 | 831053 |
| 13 | TS=(behavio?r* NEAR/1 chang*) | 21580 |
| 14 | TS=(risk and (movement? or ergonomic* or posture?)) | 18871 |
| 15 | TS=pacing | 299965 |
| 16 | TS=(worka* or worke* or workg* or worki* or workl* or workp* or work capacity or work disabilit* or work abilit* or at work or work exposure or work related or workers or job* or employee or staff or personnel or occupation or occupations or occupational or outdoor work* or day shift* or night shift* or shift work* or vocational rehabilitation or sick leave or absenteeism or sickness absen* or absente* or presente* or "return to work" or vocational reintegration or employment or unemployed or unemployment or work status or industries or industrial sector or repetitive work or wet work or (disability NEAR/1 (assess* or evaluation))) | 5650708 |
| 17 | (#13 OR #14 OR #15) AND #16 | 56675 |
| 18 | #12 OR #17 | 875535 |
| 19 | TS=(musculoskeletal or foot or feet or ankle? or knee? or hip? or coxa or limb? or low back or back pain or shoulder? or spine or spinal or elbow? or wrist? or hand? or neck) | 1104616 |
| 20 | TS=(prevention or risk or epidemiology or etiolog*) | 4147642 |
| 21 | #18 AND #19 AND #20 | 14741 |
| 22 | Refined by: WEB OF SCIENCE INDEX: ( WOS.ISTP OR WOS.ISSHP ) | 326 |
|  |  |  |


**APPENDIX C: TOPIC LIST PER CATEGORY**

| **A conceptual framework for Individual Working Practice to prevent work-related musculoskeletal disorders: a scoping review** | | |  |
| --- | --- | --- | --- |
| **2021** |  |  |  |
| **Bert van de Wijdeven** | | |  |
| **TOPICS SORTED BY CATEGORIES** | | |  |
| **WORKPLACE ADJUSTMENT (160 topics)** | | |  |
| 1 | 1 | fit workplace to their anthropometric values | (Aaras,2005) |
| 1 | 2 | adjusting distances of the screen and documents in relation to the operators | (Aaras,2005) |
| 1 | 3 | support forearms on the table top | (Aaras,2005) |
| 1 | 4 | is and adjustments of workstations | (Abareshi, 2015) |
| 1 | 5 | correction of eye-monitor distance | (Bakhtiar, 2003) |
| 1 | 6 | correction of table levels | (Bakhtiar, 2003) |
| 1 | 7 | correct placement of monitor | (Bakhtiar, 2003) |
| 1 | 8 | correction of chair height | (Bakhtiar, 2003) |
| 1 | 9 | correction of keyboard levels | (Bakhtiar, 2003) |
| 1 | 10 | correction of chair seat levels | (Bakhtiar, 2003) |
| 1 | 11 | work surface appropriate width | (Baydur, 2016) |
| 1 | 12 | leg area wide enough | (Baydur, 2016) |
| 1 | 13 | monitor placed so the top is slightly under eye level | (Baydur, 2016) |
| 1 | 14 | monitor on comfortable viewing distance | (Baydur, 2016) |
| 1 | 15 | no glare on screen | (Baydur, 2016) |
| 1 | 16 | appropriate color- brightness and contrast | (Baydur, 2016) |
| 1 | 17 | keyboard directly in front of user and monitor | (Baydur, 2016) |
| 1 | 18 | mouse close to the keyboard at the same height | (Baydur, 2016) |
| 1 | 19 | user’s body is not forced when reaching the mouse | (Baydur, 2016) |
| 1 | 20 | monitor height | (Bernaards, 2008) |
| 1 | 21 | monitor distance | (Bernaards, 2008) |
| 1 | 22 | keyboard rotation | (Bernaards, 2008) |
| 1 | 23 | both elbows supported while typing | (Bernaards, 2008) |
| 1 | 24 | elbow and forearm supported while working with mouse | (Bernaards, 2008) |
| 1 | 25 | rotation of computer worker in relationship to the position of the computer screen | (Bernaards, 2008) |
| 1 | 26 | back completely supported | (Bernaards, 2008) |
| 1 | 27 | desk/keyboard height | (Bernaards, 2008) |
| 1 | 28 | keyboard tilt | (Bernaards, 2008) |
| 1 | 29 | workstation adjustments | (Bulduk, 1997) |
| 1 | 30 | the bed will be positioned such that all participants can grasp the draw sheet under the patient with knees slightly bent and spine in a neutral position | (Callihan, 2021) |
| 1 | 31 | chair adjustments | (Dainoff, 2005) |
| 1 | 32 | work surfaces adjustments | (Dainoff, 2005) |
| 1 | 33 | correct keyboard adjustments | (Dainoff, 2005) |
| 1 | 34 | correct monitor height | (Dainoff, 2005) |
| 1 | 35 | adjustable footstools | (Dainoff, 2005) |
| 1 | 36 | ergonomics | (Dale, 2016) |
| 1 | 37 | principles of ergonomics | (Dalkilinc, 2002) |
| 1 | 38 | mouse position | (Dennerlein, 2006) |
| 1 | 39 | adjust workstation height | (Engelen, 2019) |
| 1 | 40 | safe ergonomic set ups while sitting and standing | (Engelen, 2019) |
| 1 | 41 | workstation adjustments | (Esmaeilzadeh, 2014) |
| 1 | 42 | key below elbow height | (Gerr, 2005) |
| 1 | 43 | keyboard distance | (Gerr, 2005) |
| 1 | 44 | mouse position | (Gerr, 2005) |
| 1 | 45 | chair adjustment | (Gerr, 2005) |
| 1 | 46 | eye height level with top of monitor screen | (Gerr, 2005) |
| 1 | 47 | keyboard shoulder flexion | (Gerr, 2005) |
| 1 | 48 | keyboard shoulder abduction | (Gerr, 2005) |
| 1 | 49 | keyboard wrist extension | (Gerr, 2005) |
| 1 | 50 | correct monitor and table position | (Giagio, 2019) |
| 1 | 51 | 18” distance away from the computer screen | (Goodman, 2005) |
| 1 | 52 | keyboard placement | (Goodman, 2005) |
| 1 | 53 | mouse placement | (Goodman, 2005) |
| 1 | 54 | properties of a good chair | (Goodman, 2005) |
| 1 | 55 | location of the printer- scanner- and telephone | (Goodman, 2005) |
| 1 | 56 | alternatives to the typical telephone | (Goodman, 2005) |
| 1 | 57 | lower back is supported by the chair in a slightly reclined posture with the hip flexion angle between 90–110 degrees | (Gravina, 2007) |
| 1 | 58 | workstation design | (Greene, 2005) |
| 1 | 59 | screen position | (Greene, 2005) |
| 1 | 60 | working level | (Hakkanen, 1997) |
| 1 | 61 | adjustment of the height and back support section of a bed | (Iwakiri, 2018) |
| 1 | 62 | the viewing distance should be between 3 8 and 62 cm | (Jamjumrus, 2008) |
| 1 | 63 | adjust the height of the bed | (Koshy, 2020) |
| 1 | 64 | right position of the bed | (Koshy, 2020) |
| 1 | 65 | the preferred heights of the chair | (Levanon, 2012) |
| 1 | 66 | the preferred heights of the table | (Levanon, 2012) |
| 1 | 67 | position keyboard | (Levanon, 2012) |
| 1 | 68 | the preferred heights of the screen | (Levanon, 2012) |
| 1 | 69 | workstation adjustments | (Lewis, 2001) |
| 1 | 70 | support of the arms during keyboard work | (Lindegard, 2003) |
| 1 | 71 | support of the mouse operating arm during input device work | (Lindegard, 2003) |
| 1 | 72 | bed/chair heights are routinely adjusted to reduce bending | (Lynch, 2000) |
| 1 | 73 | transfer surfaces are approximately same height | (Lynch, 2000) |
| 1 | 74 | transfer surfaces are positioned close together | (Lynch, 2000) |
| 1 | 75 | the use of monitor -distance | (Mahmud, 2015) |
| 1 | 76 | the use of keyboard -height | (Mahmud, 2015) |
| 1 | 77 | the use of mouse- close to keyboard | (Mahmud, 2015) |
| 1 | 78 | the use of chair | (Mahmud, 2015) |
| 1 | 79 | and the use of desk monitor is at arm’s length away from user | (Mahmud, 2015) |
| 1 | 80 | work tool adjustment | (Major, 2015) |
| 1 | 81 | workstation layout | (Major, 2015) |
| 1 | 82 | position of frequently used office equipment | (Marcoux, 2000) |
| 1 | 83 | adjustment of office equipment | (Marcoux, 2000) |
| 1 | 84 | the placement of the copystand | (Marcoux, 2000) |
| 1 | 85 | changing the angle of the back of your chair | (Marcoux, 2000) |
| 1 | 86 | changing the tilt of your seat | (Marcoux, 2000) |
| 1 | 87 | adjust the height of your keyboard tray | (Marcoux, 2000) |
| 1 | 88 | adjust the monitor | (Marcoux, 2000) |
| 1 | 89 | proper positioning of office supplies | (Martin, 2003) |
| 1 | 90 | adjusting chairs | (Martin, 2003) |
| 1 | 91 | placing frequently used objects within easy reach of the worker | (Martin, 2003) |
| 1 | 92 | forearms parallel-both arms from elbow to wrist parallel to the floor | (McCann, 1996) |
| 1 | 93 | table settings -height-depth-width | (Meinert, 2013) |
| 1 | 94 | lightning settings | (Meinert, 2013) |
| 1 | 95 | monitor position -height-depth-width- inclination relative to vertical | (Meinert, 2013) |
| 1 | 96 | proper positioning and adjustment of chair | (Mirmohammadi, 2012) |
| 1 | 97 | proper positioning and adjustment of desk | (Mirmohammadi, 2012) |
| 1 | 98 | proper positioning and adjustment of monitor | (Mirmohammadi, 2012) |
| 1 | 99 | proper positioning and adjustment of keyboard | (Mirmohammadi, 2012) |
| 1 | 100 | proper positioning and adjustment of mouse | (Mirmohammadi, 2012) |
| 1 | 101 | adjusting the postural components of the workstation correctly | (Montreuil, 2006) |
| 1 | 102 | adjusting the visual components of the workstation correctly | (Montreuil, 2006) |
| 1 | 103 | adjusting bed height | (Nelson, 2003) |
| 1 | 104 | have the right equipment conveniently located | (Nelson, 2006) |
| 1 | 105 | chair height | (Pillastrini, 2010) |
| 1 | 106 | desk height | (Pillastrini, 2010) |
| 1 | 107 | backrest inclination | (Pillastrini, 2010) |
| 1 | 108 | screen height | (Pillastrini, 2010) |
| 1 | 109 | screen inclination | (Pillastrini, 2010) |
| 1 | 110 | screen orientation |  |
| 1 | 111 | mouse location | (Pillastrini, 2010) |
| 1 | 112 | keyboard inclination | (Pillastrini, 2010) |
| 1 | 113 | keyboard location | (Pillastrini, 2010) |
| 1 | 114 | use of lumbar supports | (Pillastrini, 2010) |
| 1 | 115 | suitable arrangement of workstation and equipment | (Rasoulzadeh, 2012) |
| 1 | 116 | adjusting distance between monitor and user | (Rasoulzadeh, 2012) |
| 1 | 117 | adjusting seat height | (Rasoulzadeh, 2012) |
| 1 | 118 | adjusting monitor vertical and horizontal angles | (Rasoulzadeh, 2012) |
| 1 | 119 | adjusting copy holder situation | (Rasoulzadeh, 2012) |
| 1 | 120 | adjusting keyboard height | (Rasoulzadeh, 2012) |
| 1 | 121 | adjusting backrest angle | (Rasoulzadeh, 2012) |
| 1 | 122 | adjusting mouse situation | (Rasoulzadeh, 2012) |
| 1 | 123 | adjusting the intravenous infusion stand to the height of the nurse | (Ratzon, 2016) |
| 1 | 124 | widening the space between cribs in the nursery to allow improved body position when taking care of babies | (Ratzon, 2016) |
| 1 | 125 | lowering the storage position of heavy equipment | (Ratzon, 2016) |
| 1 | 126 | arrange the workstation to maximize the “comfort zone” | (Robertson, 2013) |
| 1 | 127 | reducing visual discomfort | (Robertson, 2013) |
| 1 | 128 | chair height | (Sanaeinasab, 2018) |
| 1 | 129 | pan depth | (Sanaeinasab, 2018) |
| 1 | 130 | arm rest | (Sanaeinasab, 2018) |
| 1 | 131 | back support | (Sanaeinasab, 2018) |
| 1 | 132 | adjusting chair and desk height | (Shuai, 2014) |
| 1 | 133 | adjusting backrest inclination | (Shuai, 2014) |
| 1 | 134 | adjusting keyboard inclination | (Shuai, 2014) |
| 1 | 135 | adjusting keyboard location | (Shuai, 2014) |
| 1 | 136 | adjusting screen height | (Shuai, 2014) |
| 1 | 137 | adjusting screen inclination | (Shuai, 2014) |
| 1 | 138 | back is fully supported with appropriate lumbar support | (Sigurdsson, 2011) |
| 1 | 139 | thighs and hips are supported and generally parallel to the floor | (Sigurdsson, 2011) |
| 1 | 140 | knees are about the same height as the hips with the feet slightly forward | (Sigurdsson, 2011) |
| 1 | 141 | make small adjustments to the chair or backrest position | (Sigurdsson, 2011) |
| 1 | 142 | place document holders appropriately | (Sigurdsson, 2011) |
| 1 | 143 | proper workplace adjustment | (So, 2019) |
| 1 | 144 | modification work equipment | (So, 2019) |
| 1 | 145 | ergonomic adjustments for the keyboard | (Street, 2003) |
| 1 | 146 | ergonomic adjustments for the monitor | (Street, 2003) |
| 1 | 147 | ergonomic adjustments for the chair | (Street, 2003) |
| 1 | 148 | ergonomic adjustments for the lighting | (Street, 2003) |
| 1 | 149 | ergonomic adjustments for the mouse/input devices | (Street, 2003) |
| 1 | 150 | adjusting chair height | (Taieb-Maimon, 2012) |
| 1 | 151 | adjusting desk height | (Taieb-Maimon, 2012) |
| 1 | 152 | adjusting backrest inclination | (Taieb-Maimon, 2012) |
| 1 | 153 | adjusting keyboard inclination and location | (Taieb-Maimon, 2012) |
| 1 | 154 | adjusting screen height-inclination-orientation | (Taieb-Maimon, 2012) |
| 1 | 155 | back is fully supported with appropriate lumbar support | (Yuan, 2015) |
| 1 | 156 | thighs and hips are supported and generally parallel to the floor | (Yuan, 2015) |
| 1 | 157 | knees are about the same height as the hips with the feet slightly forward | (Yuan, 2015) |
| 1 | 158 | make small adjustments to the chair or backrest position | (Yuan, 2015) |
| 1 | 159 | place document holders appropriately | (Yuan, 2015) |
| 1 | 160 | equipment placement status -the eye-monitor distance- monitor position-keyboard position-position of the mouse | (Tuncez, 2020) |
| **VARIATION (68 topics)** | | |  |
| 2 | 1 | avoid static muscle load by varying their work posture | (Aaras,2005) |
| 2 | 2 | alternation between hands | (Ackland, 2005) |
| 2 | 3 | exchange role from one intervention to the other -facilitating recovery | (Arial, 2014) |
| 2 | 4 | sitting for long periods should be avoid | (Baydur, 2016) |
| 2 | 5 | 1-2 minutes break every 20-30 minutes | (Baydur, 2016) |
| 2 | 6 | 5-10 minutes break every 1-2 hours | (Baydur, 2016) |
| 2 | 7 | use of sufficient breaks during computer work | (Bernaards, 2008) |
| 2 | 8 | suitable warm-up exercises at the beginning and in between execution of work | (Bulduk, 1997) |
| 2 | 9 | improve the duration of awkward postures | (Burford, 2017) |
| 2 | 10 | avoidance of prolonged use of muscles or the holding of the joints in one position for undue periods of time | (Dortch , 1990) |
| 2 | 11 | work-rest schedules | (Droeze, 2005) |
| 2 | 12 | variety | (Droeze, 2005) |
| 2 | 13 | to stand at certain intervals throughout the day | (Engelen, 2019) |
| 2 | 14 | moving more and sitting less | (Engelen, 2019) |
| 2 | 15 | use the sit-stand desk correctly and safely | (Engelen, 2019) |
| 2 | 16 | gradually increase standing time to prevent injury and fatigue | (Engelen, 2019) |
| 2 | 17 | take frequent breaks | (Fisher, 2015) |
| 2 | 18 | alternatives to use of a mouse | (Goodman, 2005) |
| 2 | 19 | rest breaks five minutes of every hour | (Goodman, 2005) |
| 2 | 20 | microbreaks | (Greene, 2005) |
| 2 | 21 | microbreaks with exercises | (Hallbeck, 2017) |
| 2 | 22 | recognizing strain in common areas and taking breaks when needed | (Khan, 2020) |
| 2 | 23 | time spent in non-neutral trunk and shoulder postures | (King, 2019) |
| 2 | 24 | use stoppers or brakes | (Koshy, 2020) |
| 2 | 25 | mini breaks: muscle relaxation for 1–2 seconds every 5 minutes | (Levanon, 2012) |
| 2 | 26 | breaks: stop working for 1–2 minutes every 30 minutes and for 5 minutes every hour | (Levanon, 2012) |
| 2 | 27 | take a break | (Mahmud, 2015) |
| 2 | 28 | relocation | (Major, 2015) |
| 2 | 29 | job rotation | (Major, 2015) |
| 2 | 30 | taking posture breaks | (Marcoux, 2000) |
| 2 | 31 | change of body position | (Marcoux, 2000) |
| 2 | 32 | switching the finger pressing the button | (Marcoux, 2000) |
| 2 | 33 | alternate in operating the trackball-mouse | (Marcoux, 2000) |
| 2 | 34 | operating the mouse with the non-dominant hand | (Marcoux, 2000) |
| 2 | 35 | use of varied postures | (Martin, 2003) |
| 2 | 36 | alternate heavy and light work tasks | (Martin, 2003) |
| 2 | 37 | changing positions throughout the day | (Martin, 2003) |
| 2 | 38 | utilize alternative ways of completing job duties | (Martin, 2003) |
| 2 | 39 | take breaks | (McGuckin, 2017) |
| 2 | 40 | alternate workposition -standing-sitting-walking | (McGuckin, 2017) |
| 2 | 41 | the frequency of positioning | (Ouellet, 2014) |
| 2 | 42 | tension-relaxation ratio in sequence | (Petersen, 1980) |
| 2 | 43 | ‘‘micro breaks’’ | (Pillastrini, 2010) |
| 2 | 44 | adjusting work–rest scheduling | (Rasoulzadeh, 2012) |
| 2 | 45 | varying work postures -sitting and standing | (Robertson, 2013) |
| 2 | 46 | taking micro-rest breaks | (Robertson, 2013) |
| 2 | 47 | duration of sitting | (Sanaeinasab, 2018) |
| 2 | 48 | doing breaks | (Shuai, 2014) |
| 2 | 49 | stand up and walk around for a few minutes periodically | (Sigurdsson, 2011) |
| 2 | 50 | perform some of your tasks in standing -computing-reading-phone-meeting | (Sigurdsson, 2011) |
| 2 | 51 | take rest breaks | (Sigurdsson, 2011) |
| 2 | 52 | introduce variety into the tasks -mouse/keyboard | (Sigurdsson, 2011) |
| 2 | 53 | sitting/standing | (Sigurdsson, 2011) |
| 2 | 54 | making movement | (Street, 2003) |
| 2 | 55 | taking breaks | (Street, 2003) |
| 2 | 56 | taking breaks | (Taieb-Maimon, 2012) |
| 2 | 57 | have a break | (Taieb-Maimon, 2012) |
| 2 | 58 | change position | (Taieb-Maimon, 2012) |
| 2 | 59 | duration in seated position | (Toomingas, 2012) |
| 2 | 60 | changing position standing/sitting | (Toomingas, 2012) |
| 2 | 61 | stand up and walk around for a few minutes periodically | (Yuan, 2015) |
| 2 | 62 | perform some of your tasks in standing -computing-reading-phone-meeting | (Yuan, 2015) |
| 2 | 63 | take rest breaks | (Yuan, 2015) |
| 2 | 64 | introduce variety into the tasks -mouse/keyboard | (Yuan, 2015) |
| 2 | 65 | sitting/standing | (Yuan, 2015) |
| 2 | 66 | take rest breaks | (Yuan, 2015) |
| 2 | 67 | when carrying containers with one hand: alternate hands | (Yuan, 2015) |
| 2 | 68 | importance of breaks during the work hours | (Tuncez, 2020) |
| **EXERCISING (56 topics)** | | |  |
| 3 | 1 | information on suitable stretching exercises | (Abareshi, 2015) |
| 3 | 2 | general physical activity | (Arnason,2018) |
| 3 | 3 | optimal body alinement through a variety of physical exercises | (Arnason,2018) |
| 3 | 4 | specific shoulder and thoracic strengthening | (Arnason,2018) |
| 3 | 5 | mobility exercises | (Arnason,2018) |
| 3 | 6 | warm-up routines | (Arnason,2018) |
| 3 | 7 | relaxing the neck muscles while working for long hours | (Bakhtiar, 2003) |
| 3 | 8 | individual based endurance and musculoskeletal strengthening exercise programmes | (Bakhtiar, 2003) |
| 3 | 9 | posture correction exercises | (Bakhtiar, 2003) |
| 3 | 10 | neck stretching and strengthening exercises | (Bakhtiar, 2003) |
| 3 | 11 | twice a day office-exercising | (Baydur, 2016) |
| 3 | 12 | core activation must be used throughout each of the movement patterns | (Callihan, 2021) |
| 3 | 13 | basic exercise | (Dalkilinc, 2002) |
| 3 | 14 | physical fitness | (Dalkilinc, 2002) |
| 3 | 15 | office exercise | (Dalkilinc, 2002) |
| 3 | 16 | appropriate stretching -warm-up techniques | (Daynard, 2001) |
| 3 | 17 | training of the body -important when static work posture is part of the job | (Droeze, 2005) |
| 3 | 18 | workplace exercises | (Esmaeilzadeh, 2014) |
| 3 | 19 | daily stretching and strengthening routine | (Feldstein, 1993) |
| 3 | 20 | apply a progressive musculoskeletal relaxation technique | (Fisher, 2015) |
| 3 | 21 | specific exercises to be performed before and after the surgical procedures-nonresistance active exercises | (Giagio, 2019) |
| 3 | 22 | exercise performed during the rest breaks | (Goodman, 2005) |
| 3 | 23 | eye exercises | (Goodman, 2005) |
| 3 | 24 | exercise | (Greene, 2005) |
| 3 | 25 | patient handling exercises in case of inactive patients | (Jaromi, 2018) |
| 3 | 26 | patient handling exercises in case of partially active patients | (Jaromi, 2018) |
| 3 | 27 | static stabilization exercises | (Jaromi, 2018) |
| 3 | 28 | elongation exercises | (Jaromi, 2018) |
| 3 | 29 | isometric trunk muscle strengthening exercises in lying position | (Jaromi, 2018) |
| 3 | 30 | exercises for correct body posture | (Jaromi, 2018) |
| 3 | 31 | isometric muscle strengthening exercises in sitting position | (Jaromi, 2018) |
| 3 | 32 | relaxation and breathing exercises | (Jaromi, 2018) |
| 3 | 33 | exercises for trunk strengthening | (Jaromi, 2018) |
| 3 | 34 | stretching exercises | (Jaromi, 2018) |
| 3 | 35 | dynamic stabilization exercises | (Jaromi, 2018) |
| 3 | 36 | progressive strength training on the basis of biomechanics | (Jaromi, 2018) |
| 3 | 37 | trunk muscle strengthening exercises in yoga and pilates | (Jaromi, 2018) |
| 3 | 38 | trunk muscle strengthening exercises in fitness | (Jaromi, 2018) |
| 3 | 39 | low-impact aerobic | (Jaromi, 2018) |
| 3 | 40 | stretching and eccentric exercises twice a day for the upper limbs | (Levanon, 2012) |
| 3 | 41 | stretching and breaks every 2 h during vdt use | (Lewis, 2001) |
| 3 | 42 | stretching exercises | (Marcoux, 2000) |
| 3 | 43 | stress relieving activities | (Marcoux, 2000) |
| 3 | 44 | the use of relevant stretches throughout the workday | (Martin, 2003) |
| 3 | 45 | the application of segmental stabilizing exercises | (Ratzon, 2016) |
| 3 | 46 | stretching exercises | (Sanaeinasab, 2018) |
| 3 | 47 | performing regular exercises to strengthen back and shoulder muscles | (Sezgin, 2018) |
| 3 | 48 | doing exercises while in the office | (Shuai, 2014) |
| 3 | 49 | stretch the fingers-hands-arms-torso | (Sigurdsson, 2011) |
| 3 | 50 | making exercises -stretching | (Street, 2003) |
| 3 | 51 | doing exercises while in the office | (Taieb-Maimon, 2012) |
| 3 | 52 | do stretching | (Taieb-Maimon, 2012) |
| 3 | 53 | stretch the fingers-hands-arms-torso | (Yuan, 2015) |
| 3 | 54 | use stretching | (Yuan, 2015) |
| 3 | 55 | 20-min morning exercise sessions performed before the start of daily work | (Karimi, 2020) |
| 3 | 56 | exercises to adopt a neutral back and help to fit the body | (Sene-Mir, 2020) |
| **USE OF AIDS** | | |  |
| 4 | 1 | using optical tool | (Aghilinejad, 2016) |
| 4 | 2 | ceiling lift usage | (Alamgir, 2011) |
| 4 | 3 | using stairway evacuation chair instead of stretcher to carry the patient in stairway | (Arial, 2014) |
| 4 | 4 | using hydraulic lifting devices when available -e.g. devices lifting and lowering the cot into and out of the ambulance | (Arial, 2014) |
| 4 | 5 | use a backpack type of emergency bag to carry heavy material -e.g. oxygen bottle | (Arial, 2014) |
| 4 | 6 | add a moveable step or a running board to the emergency vehicle to ease the access of the patient with minimal support | (Arial, 2014) |
| 4 | 7 | use a hard slide board | (Callihan, 2021) |
| 4 | 8 | use equipment | (Carta, 2010) |
| 4 | 9 | use of document holder | (Dainoff, 2005) |
| 4 | 10 | using the available tools/equipment | (Dale, 2016) |
| 4 | 11 | use of equipment -transfer belts-sliding devices-total body mechanical lifts | (Daynard, 2001) |
| 4 | 12 | the type of assistive equipment that should be used | (Daynard, 2001) |
| 4 | 13 | use of tools | (Denadai, 2021) |
| 4 | 14 | use equipment | (Dennerlein, 2017) |
| 4 | 15 | use lifting device | (Dennerlein, 2017) |
| 4 | 16 | use of the patient- and dentist chair | (Droeze, 2005) |
| 4 | 17 | proper use of equipment to aid in transfer maneuvers | (Feldstein, 1993) |
| 4 | 18 | armrest | (Gerr, 2005) |
| 4 | 19 | keyboard wrist rest | (Gerr, 2005) |
| 4 | 20 | use of foot pedal | (Giagio, 2019) |
| 4 | 21 | placing and use of the equipment-the surgical team-lead apron-antifatigue mat- footwear-footrests-sit/stand stool | (Giagio, 2019) |
| 4 | 22 | footrest- wrist- forearm supports | (Goodman, 2005) |
| 4 | 23 | use document holders | (Goodman, 2005) |
| 4 | 24 | transfer boards | (Lynch, 2000) |
| 4 | 25 | walking belts are used for patient transfers | (Lynch, 2000) |
| 4 | 26 | transfer systems are used for bed/chair patient moves | (Lynch, 2000) |
| 4 | 27 | lift hoists are used for immobile patients | (Lynch, 2000) |
| 4 | 28 | workstation equipment | (Major, 2015) |
| 4 | 29 | use of a headset/speaker phone | (Marcoux, 2000) |
| 4 | 30 | proper use of office supplies | (Martin, 2003) |
| 4 | 31 | use of ergonomic equipment | (Martin, 2003) |
| 4 | 32 | use special equipment -adjustable keyboard trays-lumbar supports- document holders- footrests- monitor risers- wrist rests- glare screen- organizer | (Martin, 2003) |
| 4 | 33 | change equipment -cordless phones | (McGuckin, 2017) |
| 4 | 34 | using air matrass | (Nelson, 2003) |
| 4 | 35 | use friction reducing device | (Nelson, 2003) |
| 4 | 36 | using chair that facilitates lateral transfer | (Nelson, 2003) |
| 4 | 37 | using lift | (Nelson, 2003) |
| 4 | 38 | using sling | (Nelson, 2003) |
| 4 | 39 | use special equipment -slings-gait belts-sliding boards- | (Nelson, 2006) |
| 4 | 40 | use lift-devices | (Nelson, 2006) |
| 4 | 41 | us of a lumbar cushion | (Pillastrini, 2010) |
| 4 | 42 | us of a a gel mouse-pad with ergonomic wrist support and | (Pillastrini, 2010) |
| 4 | 43 | us of a foot rest | (Pillastrini, 2010) |
| 4 | 44 | us of a paper mount | (Pillastrini, 2010) |
| 4 | 45 | the use of aid accessories | (Ratzon, 2016) |
| 4 | 46 | use forearm supports | (Shuai, 2014) |
| 4 | 47 | use foot rests | (Shuai, 2014) |
| 4 | 48 | use wrist/palm supports while using a computer mouse | (Sigurdsson, 2011) |
| 4 | 49 | apply forearm supports as needed | (Taieb-Maimon, 2012) |
| 4 | 50 | apply foot rests as needed | (Taieb-Maimon, 2012) |
| 4 | 51 | use trolleys to transport | (Wu, 2009) |
| 4 | 52 | use of total body lift | (Yassi, 2001) |
| 4 | 53 | use of transfer belts | (Yassi, 2001) |
| 4 | 54 | use of small sliding devices | (Yassi, 2001) |
| 4 | 55 | use of mechanical patient lift | (Yassi, 2001) |
| 4 | 56 | use of sit-stand lifts | (Yassi, 2001) |
| 4 | 57 | use wrist/palm supports while using a computer mouse | (Yuan, 2015) |
| 4 | 58 | use equipment if available | (Yuan, 2015) |
| **PROFESSIONAL SKILLS (53 topics)** | | |  |
| 5 | 1 | during transfer to the hospital: pushing leg against the stretcher | (Arial, 2014) |
| 5 | 2 | inserting the foot in the stretcher’s frame during transfer to the hospital | (Arial, 2014) |
| 5 | 3 | protecting the patient’s bed permitting to put boots on bed-allowing better adapted postures | (Arial, 2014) |
| 5 | 4 | leaning and sliding the shoulder against the wall while carrying a stretcher in stairways | (Arial, 2014) |
| 5 | 5 | grip forces | (Dempsey, 2004) |
| 5 | 6 | cutting moments | (Dempsey, 2004) |
| 5 | 7 | deboner skills | (Dempsey, 2004) |
| 5 | 8 | tool handling | (Droeze, 2005) |
| 5 | 9 | do not pass the probe cable around the back of the neck | (Fisher, 2015) |
| 5 | 10 | position the patient as close as possible to the operator | (Fisher, 2015) |
| 5 | 11 | place the scanning arm on top of the patient | (Fisher, 2015) |
| 5 | 12 | hold the transducer with a palmar grip | (Fisher, 2015) |
| 5 | 13 | type of tool | (Hakkanen, 1997) |
| 5 | 14 | the proper usage of the welfare equipment -hoist - sliding board or a sliding sheet- automatic bathing equipment | (Iwakiri, 2018) |
| 5 | 15 | patient handling according to the method of dotte, | (Jaromi, 2018) |
| 5 | 16 | patient handling according to the method of bobath | (Jaromi, 2018) |
| 5 | 17 | indirect vision skills | (Jeong, 2020) |
| 5 | 18 | optimal bed positioning | (Khan, 2020) |
| 5 | 19 | optimal patient positioning | (Khan, 2020) |
| 5 | 20 | optimal monitor positioning maintenance of a neutral body position | (Khan, 2020) |
| 5 | 21 | workingtechnique -lower clothing-assist client to sit-assist client to stand-posterior perineal care- replace clothing | (King, 2019) |
| 5 | 22 | reducing force exerted on the keyboard | (Levanon, 2012) |
| 5 | 23 | type of working technique during input device work | (Lindegard, 2003) |
| 5 | 24 | using control strategies including engineering controls -walking belts | (Lynch, 2000) |
| 5 | 25 | equipment is in proper working order and properly maintained | (Lynch, 2000) |
| 5 | 26 | work tool use | (Major, 2015) |
| 5 | 27 | proper usage of keyboard | (Mirmohammadi, 2012) |
| 5 | 28 | use algorithms to support decision making | (Nelson, 2006) |
| 5 | 29 | decide which equipment to use | (Nelson, 2006) |
| 5 | 30 | know how to use the equipment | (Nelson, 2006) |
| 5 | 31 | the position of the piece of meat in relation to the worker | (Ouellet, 2014) |
| 5 | 32 | type of grip on the knife in relation to step | (Ouellet, 2014) |
| 5 | 33 | the way of variation of the grip | (Ouellet, 2014) |
| 5 | 34 | direction of the cut | (Ouellet, 2014) |
| 5 | 35 | the angle of the knife | (Ouellet, 2014) |
| 5 | 36 | the direction of movement | (Ouellet, 2014) |
| 5 | 37 | number and length of knife strokes | (Ouellet, 2014) |
| 5 | 38 | the parts of the bone -zones where the knife thrusts are found | (Ouellet, 2014) |
| 5 | 39 | arranging their workstation to support their work needs | (Robertson, 2013) |
| 5 | 40 | using supportive equipment properly | (Sezgin, 2018) |
| 5 | 41 | apply effective work practices | (Sigurdsson, 2011) |
| 5 | 42 | route selection | (Tiemessen, 2009) |
| 5 | 43 | driving speed | (Tiemessen, 2009) |
| 5 | 44 | driving style | (Tiemessen, 2009) |
| 5 | 45 | apply effective work practices | (Yuan, 2015) |
| 5 | 46 | check for tags on loads | (Yuan, 2015) |
| 5 | 47 | before lifting- always test the load for stability and weight | (Yuan, 2015) |
| 5 | 48 | choose the size of gloves that fits properly | (Yuan, 2015) |
| 5 | 49 | get a secure grip | (Yuan, 2015) |
| 5 | 50 | support the container on or against a fixed object-rack-or stand while pouring the contents | (Yuan, 2015) |
| 5 | 51 | clearance for hand-finger | (Zare, 2018) |
| 5 | 52 | workspace for hands | (Zare, 2018) |
| 5 | 53 | tightening torque | (Zare, 2018) |
| **PROFESSIONAL MANNERS (86 topices)** | | |  |
| 6 | 1 | asking the driver to stop the ambulance e.g. performing an intravenous injection | (Arial, 2014) |
| 6 | 2 | requiring support from firefighters with appropriate lifting equipment when needed | (Arial, 2014) |
| 6 | 3 | moving the furniture to have more space around the patient -and adopt more comfortable postures- | (Arial, 2014) |
| 6 | 4 | put emergency bags on a table beside of the patient’s bed -access to it with little bending and twisting of the trunk | (Arial, 2014) |
| 6 | 5 | remove frames from wall and use nail to hang intravenous saline bag | (Arial, 2014) |
| 6 | 6 | asking the colleague to help with moving the patient on bed | (Arial, 2014) |
| 6 | 7 | create a counterweight by pushing on stretcher handles as the colleague pulls on the other side to lift it -stretcher with extendable handles | (Arial, 2014) |
| 6 | 8 | asking the patient to walk to the emergency vehicle -or supporting the patient to do so | (Arial, 2014) |
| 6 | 9 | using tricks of the trade to make sure the patient is not simulating unconsciousness to avoid unnecessary transport | (Arial, 2014) |
| 6 | 10 | work stress | (Bernaards, 2008) |
| 6 | 11 | over-commitment | (Bernaards, 2008) |
| 6 | 12 | by horizontal transfer: one member of the team will be on either side of the patient | (Callihan, 2021) |
| 6 | 13 | coordinate the effort | (Callihan, 2021) |
| 6 | 14 | a maximum safe and sustainable lifting weight | (Cheng, 2009) |
| 6 | 15 | patient assessment | (Daynard, 2001) |
| 6 | 16 | planning lifts ahead | (Daynard, 2001) |
| 6 | 17 | the number of people required to perform the transfer | (Daynard, 2001) |
| 6 | 18 | maintenance of knife sharpness | (Dempsey, 2004) |
| 6 | 19 | pace | (Droeze, 2005) |
| 6 | 20 | workload | (Droeze, 2005) |
| 6 | 21 | support by an assistant | (Droeze, 2005) |
| 6 | 22 | ono-on-one assistance | (Feldstein, 1993) |
| 6 | 23 | problem identification on environment hazards | (Feldstein, 1993) |
| 6 | 24 | use a chair with adjustable height | (Fisher, 2015) |
| 6 | 25 | use of the manual for the care method | (Iwakiri, 2018) |
| 6 | 26 | no lifting of residents by human power | (Iwakiri, 2018) |
| 6 | 27 | communicating and functioning effectively with team members such as holding the endoscope when 2 hands are required for maneuvering | (Khan, 2020) |
| 6 | 28 | asking assistants to reposition the patient to lessen musculoskeletal strain | (Khan, 2020) |
| 6 | 29 | obtain the patient's verbal consent | (Koshy, 2020) |
| 6 | 30 | do risk assessment | (Koshy, 2020) |
| 6 | 31 | use of a command like “go” at the time of transfer | (Koshy, 2020) |
| 6 | 32 | ask for assistance | (Koshy, 2020) |
| 6 | 33 | aisles and floors are clear of obstructions | (Lynch, 2000) |
| 6 | 34 | floors are dry and clean | (Lynch, 2000) |
| 6 | 35 | doors are opened before stretchers pushed through | (Lynch, 2000) |
| 6 | 36 | bed and chair wheels are locked before patient transfer | (Lynch, 2000) |
| 6 | 37 | work in teams to transfer patients | (Lynch, 2000) |
| 6 | 38 | provide constructive risk-reduction feedback to others | (Lynch, 2000) |
| 6 | 39 | work tool maintenance | (Major, 2015) |
| 6 | 40 | asking for help | (Major, 2015) |
| 6 | 41 | clean up footspace | (Martin, 2003) |
| 6 | 42 | apply a no lift policy | (Nelson, 2006) |
| 6 | 43 | assess the area for clutter | (Nelson, 2006) |
| 6 | 44 | assess the area for accessibility to the patient | (Nelson, 2006) |
| 6 | 45 | assess the area and availability of devices | (Nelson, 2006) |
| 6 | 46 | have the right equipment available | (Nelson, 2006) |
| 6 | 47 | have the right equipment in good working order | (Nelson, 2006) |
| 6 | 48 | plan the lift | (Nelson, 2006) |
| 6 | 49 | communicate with staff and the patient | (Nelson, 2006) |
| 6 | 50 | work together | (Nelson, 2006) |
| 6 | 51 | assess the patient’s ability to assist | (Nelson, 2006) |
| 6 | 52 | assess patient’s ability to comprehend instructions and cooperate | (Nelson, 2006) |
| 6 | 53 | assess the patient’s baseline physical and mental status before transfer | (O’Donnell, 2011) |
| 6 | 54 | enlist appropriate number and type of personnel needed to perform the transfer | (O’Donnell, 2011) |
| 6 | 55 | gather the necessary equipment to perform the transfer | (O’Donnell, 2011) |
| 6 | 56 | prepare the environment to optimize performance of the transfer | (O’Donnell, 2011) |
| 6 | 57 | communicate with the patient the why-what- how of the transfer | (O’Donnell, 2011) |
| 6 | 58 | communicate with personnel to define roles | (O’Donnell, 2011) |
| 6 | 59 | expectations and timing of the transfer | (O’Donnell, 2011) |
| 6 | 60 | reassess the patient for change in physical or mental status post-transfer event | (O’Donnell, 2011) |
| 6 | 61 | reset the patient and unit environment | (O’Donnell, 2011) |
| 6 | 62 | the order in which the steps were followed | (Ouellet, 2014) |
| 6 | 63 | the way of preventing incidents | (Ouellet, 2014) |
| 6 | 64 | limit lifting | (Owlia, 2020) |
| 6 | 65 | asking for help for difficult tasks | (Ratzon, 2016) |
| 6 | 66 | compliance with recommendations | (Toomingas, 2012) |
| 6 | 67 | never lift the patient unless you have to | (Videman, 1989) |
| 6 | 68 | consider the size and shape of the patient before handling | (Videman, 1989) |
| 6 | 69 | consider patients of dependence | (Videman, 1989) |
| 6 | 70 | consider the availability of apparatus and equipment | (Videman, 1989) |
| 6 | 71 | consider the organization of the environment | (Videman, 1989) |
| 6 | 72 | preparations for the lift | (Videman, 1989) |
| 6 | 73 | selection of technique | (Videman, 1989) |
| 6 | 74 | avoid prolonged standing without back supports | (Wu, 2009) |
| 6 | 75 | avoid lifting heavy weight | (Wu, 2009) |
| 6 | 76 | wear appropriate shoes to avoid slips- trips-or falls | (Yuan, 2015) |
| 6 | 77 | lift only as much as you can safely handle by yourself | (Yuan, 2015) |
| 6 | 78 | avoid lifting from the floor whenever possible | (Yuan, 2015) |
| 6 | 79 | if possible- try to find a co-worker of similar height to help with the lift | (Yuan, 2015) |
| 6 | 80 | avoid carrying large or bulky loads that limit or obstruct your vision | (Yuan, 2015) |
| 6 | 81 | slide-push-roll instead of carrying when appropriate | (Yuan, 2015) |
| 6 | 82 | when there is a choice: push instead of pull | (Yuan, 2015) |
| 6 | 83 | whenever appropriate- use two hands to carry containers | (Yuan, 2015) |
| 6 | 84 | get co-worker assistance when necessary-discuss your plan so you don’t have surprise movements | (Yuan, 2015) |
| 6 | 85 | plan the lift | (Yuan, 2015) |
| 6 | 86 | never exceed the maximum acceptable load of the worker | (Yuan, 2015) |
| **TASK CONTENT AND TASK ORGANIZATION (15 topics)** | | |  |
| 7 | 1 | movements requiring power or repetitive movements should be avoid | (Baydur, 2016) |
| 7 | 2 | improve and to potentially perform the task differently incorporating the newly acquired furniture | (Burford, 2017) |
| 7 | 3 | stress reduction | (Goodman, 2005) |
| 7 | 4 | work organization | (Greene, 2005) |
| 7 | 5 | number of repetition | (Hakkanen, 1997) |
| 7 | 6 | managing the pace of one's own work | (Major, 2015) |
| 7 | 7 | time-management | (Major, 2015) |
| 7 | 8 | means to alternate work tasks | (Martin, 2003) |
| 7 | 9 | task modification | (Martin, 2003) |
| 7 | 10 | organizing work activities in a preventive manner | (Montreuil, 2006) |
| 7 | 11 | temporal organization of the task -duration- cycle-intercycle-waiting –communication-knife sharpening | (Ouellet, 2014) |
| 7 | 12 | modification task organization | (So, 2019) |
| 7 | 13 | ergonomic adjustments for the task | (Street, 2003) |
| 7 | 14 | pacing the workday | (Street, 2003) |
| 7 | 15 | alternate heavy lifting or forceful exertion tasks with less physically demanding tasks | (Yuan, 2015) |
| **MOTORIC SKILLS (323 topics)** | | |  |
| 8 | 1 | more relaxed positions of the shoulders | (Aaras,2005) |
| 8 | 2 | prevent forward bending of the trunk | (Aaras,2005) |
| 8 | 3 | body mechanics | (Alamgir, 2011) |
| 8 | 4 | ergonomics | (Alamgir, 2011) |
| 8 | 5 | patient-handling techniques | (Alamgir, 2011) |
| 8 | 6 | trunk bending position left-richt-extension-flexion | (Allread, 2000) |
| 8 | 7 | trunk twist | (Allread, 2000) |
| 8 | 8 | maximum range of motion trunk | (Allread, 2000) |
| 8 | 9 | average velocity trunk | (Allread, 2000) |
| 8 | 10 | maximum velocity trunk | (Allread, 2000) |
| 8 | 11 | maximum acceleration trunk | (Allread, 2000) |
| 8 | 12 | maximum external moment trunk | (Allread, 2000) |
| 8 | 13 | using leg as counterweight when bending trunk -pendulum | (Arial, 2014) |
| 8 | 14 | from kneeling: push up with hand on one knee | (Arial, 2014) |
| 8 | 15 | correct posture while working | (Bakhtiar, 2003) |
| 8 | 16 | right use of hands and fingers | (Bakhtiar, 2003) |
| 8 | 17 | feet touch the floor horizontally | (Baydur, 2016) |
| 8 | 18 | wrist is straight and in line with underarm | (Baydur, 2016) |
| 8 | 19 | when mouse is gripped: the wrist is in straight position | (Baydur, 2016) |
| 8 | 20 | when using the mouse: forearm is fully supported and there is no contact with sharp edges | (Baydur, 2016) |
| 8 | 21 | while using the mouse: user’s hand is not in grip or contraction for a long time | (Baydur, 2016) |
| 8 | 22 | while sitting: posture is upright-back and waist reinforced-shoulders in natural position-head and neck straight-chest forward-thighs parallel to the floor | (Baydur, 2016) |
| 8 | 23 | avoid forward trunk posture | (Bazazan, 2019) |
| 8 | 24 | correct a slouched position to an upright trunk position | (Bazazan, 2019) |
| 8 | 25 | back straight | (Bernaards, 2008) |
| 8 | 26 | neck rotation | (Bernaards, 2008) |
| 8 | 27 | ulnar deviation while typing with left and/or right hand | (Bernaards, 2008) |
| 8 | 28 | ulnar deviation while working with mouse | (Bernaards, 2008) |
| 8 | 29 | shoulders position | (Bernaards, 2008) |
| 8 | 30 | using the method of manutention dotte | (Best, 1997) |
| 8 | 31 | correct working posture | (Bulduk, 1997) |
| 8 | 32 | correct load-carrying procedures | (Bulduk, 1997) |
| 8 | 33 | stand straight with feet shoulder-width apart- shoulders back- chest pushed forward | (Callihan, 2021) |
| 8 | 34 | patient positioned between feet by direct lifting | (Callihan, 2021) |
| 8 | 35 | correct lifting technique -squatting | (Callihan, 2021) |
| 8 | 36 | correct hand positioning | (Callihan, 2021) |
| 8 | 37 | correct push and pull technique | (Callihan, 2021) |
| 8 | 38 | ergonomic postures | (Carta, 2010) |
| 8 | 39 | safe patient-handling techniques | (Carta, 2010) |
| 8 | 40 | proper technique in manual materials handling at work | (Cheng, 2009) |
| 8 | 41 | the correct lifting posture | (Cheng, 2009) |
| 8 | 42 | coping strategies -such as occupational ergonomics | (Cheng, 2009) |
| 8 | 43 | accurate ergonomic positions | (Dabaghi-Tabriz, 2020) |
| 8 | 44 | posture | (Dalkilinc, 2002) |
| 8 | 45 | lifting technique | (Daynard, 2001) |
| 8 | 46 | proper posture | (Daynard, 2001) |
| 8 | 47 | maintaining proper posture | (Daynard, 2001) |
| 8 | 48 | maintaining a good base of support | (Daynard, 2001) |
| 8 | 49 | using smooth controlled movements | (Daynard, 2001) |
| 8 | 50 | ensuring one does not twist or perform unnecessary bending and reaching | (Daynard, 2001) |
| 8 | 51 | specific transfer techniques for given types of patient-handling activities | (Daynard, 2001) |
| 8 | 52 | safe working movements | (Denadai, 2021) |
| 8 | 53 | safe working postures | (Denadai, 2021) |
| 8 | 54 | shoulder position | (Dennerlein, 2006) |
| 8 | 55 | force | (Dennerlein, 2006) |
| 8 | 56 | avoidance of positions of deformity -grasp and pinch | (Dortch , 1990) |
| 8 | 57 | avoidance of external and internaj pressures and stress to joints in positions of deformity | (Dortch , 1990) |
| 8 | 58 | use of joints in their most stable anatomical and functional plane | (Dortch , 1990) |
| 8 | 59 | use of the strongest joint available for the job | (Dortch , 1990) |
| 8 | 60 | use of correct patterns of motion | (Dortch , 1990) |
| 8 | 61 | work posture | (Droeze, 2005) |
| 8 | 62 | proper body mechanics | (Feldstein, 1993) |
| 8 | 63 | specific techniques for patient transfer | (Feldstein, 1993) |
| 8 | 64 | keep their neck straight by positioning their head directly in front of the monitor | (Fisher, 2015) |
| 8 | 65 | keep the lower back firmly supported on the chair’s backrest | (Fisher, 2015) |
| 8 | 66 | keep the trunk straight in line with the axis of the screen | (Fisher, 2015) |
| 8 | 67 | keep the wrist in a neutral position | (Fisher, 2015) |
| 8 | 68 | keep the hips-knees-ankles at a 90 degree angle | (Fisher, 2015) |
| 8 | 69 | use the principles of ergonomics | (Fisher, 2015) |
| 8 | 70 | reductions of load lever arm | (Gagnon, 2003) |
| 8 | 71 | reductions of efforts/posture in torsion | (Gagnon, 2003) |
| 8 | 72 | load tilts | (Gagnon, 2003) |
| 8 | 73 | hands positioning | (Gagnon, 2003) |
| 8 | 74 | shoulders positioning | (Gagnon, 2003) |
| 8 | 75 | feet orientation | (Gagnon, 2003) |
| 8 | 76 | velocity | (Gagnon, 1992) |
| 8 | 77 | acceleration | (Gagnon, 1992) |
| 8 | 78 | knee angle | (Gagnon, 1992) |
| 8 | 79 | trunk angle | (Gagnon, 1992) |
| 8 | 80 | max trunk extension | (Gagnon, 1992) |
| 8 | 81 | head tilt angle | (Gerr, 2005) |
| 8 | 82 | head rotation | (Gerr, 2005) |
| 8 | 83 | keyboard inner elbow angle | (Gerr, 2005) |
| 8 | 84 | keyboard wrist ulnar deviation | (Gerr, 2005) |
| 8 | 85 | mouse wrist ulnar deviation | (Gerr, 2005) |
| 8 | 86 | mouse wrist extension | (Gerr, 2005) |
| 8 | 87 | the recommended postures for their work | (Gholami, 2020) |
| 8 | 88 | the proper way of handling heavy loads | (Gholami, 2020) |
| 8 | 89 | the amplitude of downwards motion of the joint centre for the sacrum | (Gilles, 2018) |
| 8 | 90 | the distances between the joint centres for the right and left toes and between those of the right and left knees | (Gilles, 2018) |
| 8 | 91 | the euler angles for flexion of the ankles- knees and hips as well as the abduction angles for the hips | (Gilles, 2018) |
| 8 | 92 | proper positioning at a computer workstation | (Goodman, 2005) |
| 8 | 93 | back- hips- knees- ankles- elbows at 90 degrees | (Goodman, 2005) |
| 8 | 94 | wrists in neutral position | (Goodman, 2005) |
| 8 | 95 | feet flat on the floor | (Goodman, 2005) |
| 8 | 96 | legs and forearms parallel to the floor | (Goodman, 2005) |
| 8 | 97 | box-thorax distance | (Goubault, 2020) |
| 8 | 98 | joint-angles | (Goubault, 2020) |
| 8 | 99 | neutral hand-wrist position | (Gravina, 2007) |
| 8 | 100 | neutral head/neck position | (Gravina, 2007) |
| 8 | 101 | shoulder position -upper arms close to body -3–4 inches and relaxed | (Gravina, 2007) |
| 8 | 102 | forearm position -not externally rotated beyond 60 degrees | (Gravina, 2007) |
| 8 | 103 | shoulders are in a neutral position–not elevated or depressed | (Gravina, 2007) |
| 8 | 104 | elbow flexion between 90–120 degrees | (Gravina, 2007) |
| 8 | 105 | knees flexion between 90–120 degrees- legs are in neutral – not crossed or internally or externally rotated | (Gravina, 2007) |
| 8 | 106 | proper body mechanics | (Greene, 2005) |
| 8 | 107 | postural alignment | (Greene, 2005) |
| 8 | 108 | wrist angle | (Greene, 2005) |
| 8 | 109 | sitting posture | (Greene, 2005) |
| 8 | 110 | wrist-position | (Hakkanen, 1997) |
| 8 | 111 | torso-position | (Hakkanen, 1997) |
| 8 | 112 | working distance | (Hakkanen, 1997) |
| 8 | 113 | lumbar posture while lifting | (Hakkanen, 1997) |
| 8 | 114 | taking a suitable posture | (Iwakiri, 2018) |
| 8 | 115 | sit with the back at an upright -or slightly reclined- position | (Jamjumrus, 2008) |
| 8 | 116 | neck flexion should not be more than 10° | (Jamjumrus, 2008) |
| 8 | 117 | shoulder flexion should not be more than 20° | (Jamjumrus, 2008) |
| 8 | 118 | elbow flexion should be about 90° | (Jamjumrus, 2008) |
| 8 | 119 | the lower arms and hands should form a straight line | (Jamjumrus, 2008) |
| 8 | 120 | the lower legs should form the right angle -90°- with the upper legs | (Jamjumrus, 2008) |
| 8 | 121 | both feet should rest comfortably on the floor | (Jamjumrus, 2008) |
| 8 | 122 | lifting techniques | (Jaromi, 2018) |
| 8 | 123 | correct spine usage | (Jaromi, 2018) |
| 8 | 124 | horizontal lifting | (Jaromi, 2018) |
| 8 | 125 | atraumatic patient handling | (Jaromi, 2018) |
| 8 | 126 | correct body posture in sitting position | (Jaromi, 2018) |
| 8 | 127 | profession-specific motions | (Jaromi, 2018) |
| 8 | 128 | head and shoulder posture | (Jeong, 2020) |
| 8 | 129 | joint angles | (Kaufman-Cohen, 2018) |
| 8 | 130 | joint velocities | (Kaufman-Cohen, 2018) |
| 8 | 131 | joint accelerations | (Kaufman-Cohen, 2018); |
| 8 | 132 | hand position and grip | (Khan, 2020) |
| 8 | 133 | correcting situations that may create unnecessary strain -such as resolving loops in the endoscope | (Khan, 2020) |
| 8 | 134 | work posture | (King, 2019) |
| 8 | 135 | position caregiver to client | (King, 2019) |
| 8 | 136 | good posture | (Koshy, 2020) |
| 8 | 137 | initial foot distance | (Lee, 2012) |
| 8 | 138 | lumbar angle | (Lee, 2012) |
| 8 | 139 | lumbar angular velocity | (Lee, 2012) |
| 8 | 140 | lumbar angular acceleration | (Lee, 2012) |
| 8 | 141 | handforce -anterior/posterior- medial/lateral- superior/inferior- | (Lee, 2014) |
| 8 | 142 | torso kinetics -lateral bending- flexion- extension-twisting | (Lee, 2014) |
| 8 | 143 | torso-kinematics -velocity | (Lee, 2014) |
| 8 | 144 | acceleration | (Lee, 2014) |
| 8 | 145 | handheights | (Lee, 2014) |
| 8 | 146 | distance | (Lee, 2014) |
| 8 | 147 | how to sit | (Levanon, 2012) |
| 8 | 148 | position of the back | (Levanon, 2012) |
| 8 | 149 | position of the shoulders | (Levanon, 2012) |
| 8 | 150 | position of the elbows | (Levanon, 2012) |
| 8 | 151 | position of the wrists | (Levanon, 2012) |
| 8 | 152 | relaxing shoulders | (Levanon, 2012) |
| 8 | 153 | avoiding extensive wrist and finger extension | (Levanon, 2012) |
| 8 | 154 | improving sitting habits | (Levanon, 2012) |
| 8 | 155 | load distribution between different joints | (Levanon, 2012) |
| 8 | 156 | improving muscle activity relaxation | (Levanon, 2012) |
| 8 | 157 | correct workstation posture | (Lewis, 2001) |
| 8 | 158 | lifting of the computer mouse | (Lindegard, 2003) |
| 8 | 159 | range of movements during input device work | (Lindegard, 2003) |
| 8 | 160 | velocity of movements during input device | (Lindegard, 2003) |
| 8 | 161 | sitting in a tense position | (Lindegard, 2003) |
| 8 | 162 | lifting the shoulders during keyboard work | (Lindegard, 2003) |
| 8 | 163 | lifting the shoulders during input device work | (Lindegard, 2003) |
| 8 | 164 | avoid prolonged back flexion »45 degrees | (Lynch, 2000) |
| 8 | 165 | avoid lifting while back bent or twisted | (Lynch, 2000) |
| 8 | 166 | bend at knees during lifting | (Lynch, 2000) |
| 8 | 167 | keep patient/objects close to the body | (Lynch, 2000) |
| 8 | 168 | carts/stretchers are pushed- not pulled | (Lynch, 2000) |
| 8 | 169 | the range of motion -rom- for the vertical displacement of the head–shoulder | (Madeleine, 2009) |
| 8 | 170 | shoulder–hip and elbow–hip relative motion | (Madeleine, 2009) |
| 8 | 171 | improving workstation practices | (Mahmud, 2015) |
| 8 | 172 | way how operations are carried out | (Major, 2015) |
| 8 | 173 | postures adopted | (Major, 2015) |
| 8 | 174 | proper sitting posture | (Marcoux, 2000) |
| 8 | 175 | body mechanics in lifting and moving | (Marcoux, 2000) |
| 8 | 176 | improperly bending to lift boxes | (Martin, 2003) |
| 8 | 177 | excessive wrist extension | (Martin, 2003) |
| 8 | 178 | use of proper body mechanics | (Martin, 2003) |
| 8 | 179 | use of proper postures | (Martin, 2003) |
| 8 | 180 | neutral head positioning | (Martin, 2003) |
| 8 | 181 | neutral neck positioning | (Martin, 2003) |
| 8 | 182 | neutral trunk positioning | (Martin, 2003) |
| 8 | 183 | neutral wrist positioning | (Martin, 2003) |
| 8 | 184 | back straight -spine at an 85-95 degrees angle to the floor | (McCann, 1996) |
| 8 | 185 | shoulders relaxes-line of shoulders not hunched upwards toward the neck or over the chest | (McCann, 1996) |
| 8 | 186 | neck aligned with back- a continuation of the spine | (McCann, 1996) |
| 8 | 187 | feet flat-heels and toes of both feet touching the floor on platform | (McCann, 1996) |
| 8 | 188 | leg not crossed | (McCann, 1996) |
| 8 | 189 | hand-wrist position-extension-neutral-flexion | (McCann, 1996) |
| 8 | 190 | the sitting position | (Meinert, 2013) |
| 8 | 191 | appropriate postures during working | (Mirmohammadi, 2012) |
| 8 | 192 | proper usage of mouse | (Mirmohammadi, 2012) |
| 8 | 193 | using special technique for pulling back patient | (Nelson, 2003) |
| 8 | 194 | adjust position | (Nelson, 2003) |
| 8 | 195 | lifting techniques | (Nussbaum, 2001) |
| 8 | 196 | good patient-handling performance | (Nussbaum, 2001) |
| 8 | 197 | perform the transfer according to ergonomic and patient safety principles | (O’Donnell, 2011) |
| 8 | 198 | angle of movement | (Ouellet, 2014) |
| 8 | 199 | the articular range of certain movements of the shoulder-elbow-wrist | (Ouellet, 2014) |
| 8 | 200 | keep load close to the body | (Owlia, 2020) |
| 8 | 201 | hinge at the hips | (Owlia, 2020) |
| 8 | 202 | avoid bending | (Owlia, 2020) |
| 8 | 203 | avoid twisting | (Owlia, 2020) |
| 8 | 204 | time spent in flexion | (Pal, 2010) |
| 8 | 205 | time spent in lateral bending. time spent in axial twist | (Pal, 2010) |
| 8 | 206 | forces on lumbar region | (Pal, 2010) |
| 8 | 207 | body posture | (Petersen, 1980) |
| 8 | 208 | arm-head position | (Petersen, 1980) |
| 8 | 209 | the preservation of physiological lumbar lordosis | (Pillastrini, 2010) |
| 8 | 210 | the distribution of the upper body weight on backrest | (Pillastrini, 2010) |
| 8 | 211 | the correct alignment of the lumbar curvature | (Pillastrini, 2010) |
| 8 | 212 | the support of the forearms on the table top | (Pillastrini, 2010) |
| 8 | 213 | foot position in relation with object while lifting | (Plamondon, 2006) |
| 8 | 214 | choise of arm use -left/right | (Plamondon, 2006) |
| 8 | 215 | moving the rod towards the body before lifting | (Plamondon, 2006) |
| 8 | 216 | the way the lower hand grasps the rod -supination/ pronation | (Plamondon, 2006) |
| 8 | 217 | the height the lower hand grasp the rod | (Plamondon, 2006) |
| 8 | 218 | horizontal distance of the hands from the l5/s1 joints | (Plamondon, 2010) |
| 8 | 219 | flexion in right and left knee | (Plamondon, 2010) |
| 8 | 220 | duration of transfer | (Plamondon, 2010) |
| 8 | 221 | path length of a box | (Plamondon, 2010) |
| 8 | 222 | upper trunk flexion range | (Plamondon, 2010) |
| 8 | 223 | maximum vertical hand distance to l5/s1 | (Plamondon, 2010) |
| 8 | 224 | minimum -shortest- distance of subject’s centre of gravity from the floor | (Plamondon, 2010) |
| 8 | 225 | avoiding rotational movements | (Ratzon, 2016) |
| 8 | 226 | avoiding awkward and uncomfortable body positions | (Ratzon, 2016) |
| 8 | 227 | working as closely as possible to the patient | (Ratzon, 2016) |
| 8 | 228 | lifting with bended knees | (Ratzon, 2016) |
| 8 | 229 | rang of motion -forward bending > 45 degrees | (Ribeiro, 2014) |
| 8 | 230 | frequency of bending >45 degrees -more than 2x per minute | (Ribeiro, 2014) |
| 8 | 231 | duration of bending >5 seconds | (Ribeiro, 2014) |
| 8 | 232 | wrist motion: pronation/supination -center-mid-extreme-out of range | (Ruff,2013) |
| 8 | 233 | wrist motion: flexion/extension -center-mid-extreme-out of range | (Ruff,2013) |
| 8 | 234 | wrist motion: abduction/adduction- center-mid-extreme-out of range | (Ruff,2013) |
| 8 | 235 | postures when using telephone | (Sanaeinasab, 2018) |
| 8 | 236 | postures when using monitor | (Sanaeinasab, 2018) |
| 8 | 237 | postures when using keyboard | (Sanaeinasab, 2018) |
| 8 | 238 | postures when using mouse | (Sanaeinasab, 2018) |
| 8 | 239 | thumb forces | (Sandlund, 2017) |
| 8 | 240 | range of motion -shoulder-elbow | (Sandlund, 2017) |
| 8 | 241 | velocity -shoulder-elbow | (Sandlund, 2017) |
| 8 | 242 | following proper body mechanics -e.g. during bending down and lifting | (Sezgin, 2018) |
| 8 | 243 | adjusting joint angles | (Shuai, 2014) |
| 8 | 244 | adjusting work posture | (Shuai, 2014) |
| 8 | 245 | hands-wrists- and forearms are straight and roughly parallel to the floor | (Sigurdsson, 2011) |
| 8 | 246 | head is forward facing- balanced and generally in-line with the torso | (Sigurdsson, 2011) |
| 8 | 247 | shoulders are relaxed | (Sigurdsson, 2011) |
| 8 | 248 | upper arms hang at the side of the body | (Sigurdsson, 2011) |
| 8 | 249 | elbows remain close to the body | (Sigurdsson, 2011) |
| 8 | 250 | elbows are bent between 90 degrees and 120 degrees | (Sigurdsson, 2011) |
| 8 | 251 | feet are fully supported by the floor or a footrest | (Sigurdsson, 2011) |
| 8 | 252 | use neutral wrist angles | (Sigurdsson, 2011) |
| 8 | 253 | the maintenance of the good head and scapula position -retraction- through -conditioning- exercises & biofeedback | (So, 2019) |
| 8 | 254 | neutral positions of the body | (Street, 2003) |
| 8 | 255 | proper seated posture | (Street, 2003) |
| 8 | 256 | safe keyboarding techniques | (Street, 2003) |
| 8 | 257 | handling technics-shoulder lift- orthodox lift- through arm lift-under arm drag | (Hollis 1981) |
| 8 | 258 | adjusting joint angles | (Taieb-Maimon, 2012) |
| 8 | 259 | adjusting work postures | (Taieb-Maimon, 2012) |
| 8 | 260 | posture at beginning of the lift | (Videman, 1989) |
| 8 | 261 | the choice of hand-grip | (Videman, 1989) |
| 8 | 262 | the loading of the back while lifting | (Videman, 1989) |
| 8 | 263 | the timing of the movement | (Videman, 1989) |
| 8 | 264 | the smoothness of the movement | (Videman, 1989) |
| 8 | 265 | avoid bending the trunk forward over 20 degrees | (Wu, 2009) |
| 8 | 266 | use lifting-related techniques for manual foup handling | (Wu, 2009) |
| 8 | 267 | avoid a starting position of lifting lower than knuckle height | (Wu, 2009) |
| 8 | 268 | avoid twisting the trunk over 45 degrees | (Wu, 2009) |
| 8 | 269 | trunk inclination holding time | (Yan, 2018) |
| 8 | 270 | trunk inclination angle | (Yan, 2018) |
| 8 | 271 | trunk inclination frequency | (Yan, 2018) |
| 8 | 272 | apply patient handling techniques | (Yassi, 2001) |
| 8 | 273 | apply a pushing technique | (Yassi, 2001) |
| 8 | 274 | avoid' a forward head posture | (Yoo, 2015) |
| 8 | 275 | avoid' a trunk flexion -=lumbar flexion angle relative to pelvis | (Yoo, 2015) |
| 8 | 276 | hands-wrists- and forearms are straight and roughly parallel to the floor | (Yuan, 2015) |
| 8 | 277 | head is forward facing- balanced and generally in-line with the torso | (Yuan, 2015) |
| 8 | 278 | shoulders are relaxed | (Yuan, 2015) |
| 8 | 279 | upper arms hang at the side of the body | (Yuan, 2015) |
| 8 | 280 | elbows remain close to the body | (Yuan, 2015) |
| 8 | 281 | elbows are bent between 90 degrees and 120 degrees | (Yuan, 2015) |
| 8 | 282 | feet are fully supported by the floor or a footrest | (Yuan, 2015) |
| 8 | 283 | use neutral wrist angles | (Yuan, 2015) |
| 8 | 284 | keep the lifts in your power zone -above the knees- below the shoulders | (Yuan, 2015) |
| 8 | 285 | use both hands whenever possible | (Yuan, 2015) |
| 8 | 286 | avoid jerking by using smooth-even motions | (Yuan, 2015) |
| 8 | 287 | keep the load as close to the body as possible | (Yuan, 2015) |
| 8 | 288 | to the extent feasible use your legs to push up and lift the load-not the upper body or back | (Yuan, 2015) |
| 8 | 289 | do not twist your body | (Yuan, 2015) |
| 8 | 290 | step to one side or the other to turn | (Yuan, 2015) |
| 8 | 291 | if you must lift from the floor do not bend at the waist | (Yuan, 2015) |
| 8 | 292 | use squat or stoop lifting | (Yuan, 2015) |
| 8 | 293 | lift the load with a smooth body motion -avoid jerking | (Yuan, 2015) |
| 8 | 294 | when turning: do not twist -turn with the feet rather than twisting of the trunk | (Yuan, 2015) |
| 8 | 295 | work posture | (Zare, 2018) |
| 8 | 296 | static back posture | (Zare, 2018) |
| 8 | 297 | static neck posture | (Zare, 2018) |
| 8 | 298 | static shoulder posture | (Zare, 2018) |
| 8 | 299 | wrist posture | (Zare, 2018) |
| 8 | 300 | two-handed lifts | (Zare, 2018) |
| 8 | 301 | one-handed lifts | (Zare, 2018) |
| 8 | 302 | pushing/pulling force—whole body | (Zare, 2018) |
| 8 | 303 | pushing/pulling with the hand-arm | (Zare, 2018) |
| 8 | 304 | pushing/pulling fingers | (Zare, 2018) |
| 8 | 305 | movement -continuous steps | (Zare, 2018) |
| 8 | 306 | climbing/stepping over | (Zare, 2018) |
| 8 | 307 | improving working postures | (Karimi, 2020) |
| 8 | 308 | reduce the upper-arm elevations by keeping the elbow close to the trunk | (Lind, 2020) |
| 8 | 309 | arrange the position of the letter trays to minimize the upper-arm elevation | (Lind, 2020) |
| 8 | 310 | arm elevation | (Lind, 2020b) |
| 8 | 311 | trunk inclination | (Lind, 2020b) |
| 8 | 312 | time spent in trunk flexion | (Porta, 2021) |
| 8 | 313 | rang of motion -forward bending > 45 degrees | (Ribeiro, 2014) |
| 8 | 314 | frequency of bending >45 degrees -more than 2x per minute | (Ribeiro, 2014) |
| 8 | 315 | duration of bending >5 seconds | (Ribeiro, 2014) |
| 8 | 316 | feet placed asymmetrically- one beside the load and the other behind it | (Sene-Mir, 2020) |
| 8 | 317 | knees in a semi-squat position -moderate flexion | (Sene-Mir, 2020) |
| 8 | 318 | a neutral back position at any front inclination of back | (Sene-Mir, 2020) |
| 8 | 319 | load placed closed to the body | (Sene-Mir, 2020) |
| 8 | 320 | arms extended or slightly flexed | (Sene-Mir, 2020) |
| 8 | 321 | do not start or finish carrying phase while lifting and lowering phases are being carried out | (Sene-Mir, 2020) |
| 8 | 322 | appropriate joint position-wrist- elbow- neck- hip-knee | (Tuncez, 2020) |
| 8 | 323 | right posture | (Tuncez, 2020) |
|  |  |  |  |
|  | 819 |  |  |
